# Cationic Arginine-Rich Peptides (CARPs): A Novel Class of Neuroprotective Agents With a Multimodal Mechanism of Action

**DOI:** 10.3389/fneur.2020.00108

**Published:** 2020-02-25

**Authors:** Bruno P. Meloni, Frank L. Mastaglia, Neville W. Knuckey

**Affiliations:** ^1^Department of Neurosurgery, QEII Medical Centre, Sir Charles Gairdner Hospital, Nedlands, WA, Australia; ^2^Perron Institute for Neurological and Translational Science, Nedlands, WA, Australia; ^3^Centre for Neuromuscular and Neurological Disorders, The University of Western Australia, Nedlands, WA, Australia

**Keywords:** cationic arginine-rich peptides, neuroprotection, cell-penetrating peptides, arginine, guanidinium head group, TAT

## Abstract

There are virtually no clinically available neuroprotective drugs for the treatment of acute and chronic neurological disorders, hence there is an urgent need for the development of new neuroprotective molecules. Cationic arginine-rich peptides (CARPs) are an expanding and relatively novel class of compounds, which possess intrinsic neuroprotective properties. Intriguingly, CARPs possess a combination of biological properties unprecedented for a neuroprotective agent including the ability to traverse cell membranes and enter the CNS, antagonize calcium influx, target mitochondria, stabilize proteins, inhibit proteolytic enzymes, induce pro-survival signaling, scavenge toxic molecules, and reduce oxidative stress as well as, having a range of anti-inflammatory, analgesic, anti-microbial, and anti-cancer actions. CARPs have also been used as carrier molecules for the delivery of other putative neuroprotective agents across the blood-brain barrier and blood-spinal cord barrier. However, there is increasing evidence that the neuroprotective efficacy of many, if not all these other agents delivered using a cationic arginine-rich cell-penetrating peptide (CCPPs) carrier (e.g., TAT) may actually be mediated largely by the properties of the carrier molecule, with overall efficacy further enhanced according to the amino acid composition of the cargo peptide, in particular its arginine content. Therefore, in reviewing the neuroprotective mechanisms of action of CARPs we also consider studies using CCPPs fused to a putative neuroprotective peptide. We review the history of CARPs in neuroprotection and discuss in detail the intrinsic biological properties that may contribute to their cytoprotective effects and their usefulness as a broad-acting class of neuroprotective drugs.

## Introduction

Despite the enormous global impact of neurological disorders and the extensive research over many decades, there is still a lack of proven clinically effective pharmacological neuroprotective therapies capable of reducing the severity of brain or spinal cord tissue injury in acute (e.g., stroke, traumatic brain injury and spinal cord injury, and hypoxic-ischemic encephalopathy) or chronic (Alzheimer's disease, Parkinson's disease, and amyotrophic lateral sclerosis) neurological disorders. The few neuroprotective treatments that are available, such as riluzole for amyotrophic lateral sclerosis and memantine for Alzheimer's disease provide only modest benefits. While hypothermia is used as a neuroprotective therapy for neonatal encephalopathy and for comatose survivors of cardiac arrest, it is difficult to implement due to the need for specialized equipment and intensive patient monitoring, and its efficacy is also limited.

Hence, the development of effective neuroprotective drugs for the treatment of a variety of neurological disorders remains an urgent priority. To make matters worse, due to past clinical failures, some researchers, physicians, and pharmaceutical companies are reluctant to continue research focused on the development of neuroprotective agents. However, most impartial observers would agree that the benefits of continuing to pursue the discovery of neuroprotective therapies far outweigh the risks. With this in mind, it is also intuitive that in order to increase the chances of achieving translational success at the clinical level, it is preferable that any new neuroprotective drug should have a multimodal mechanism of action. To this end, cationic arginine-rich peptides (CARPs) represent a relatively novel and expanding class of compounds, which possess an array of intrinsic neuroprotective properties, and are thus ideal molecules for development as therapies for a broad range of neurological disorders.

## General Aspects of CARPs

**Figure 1:**
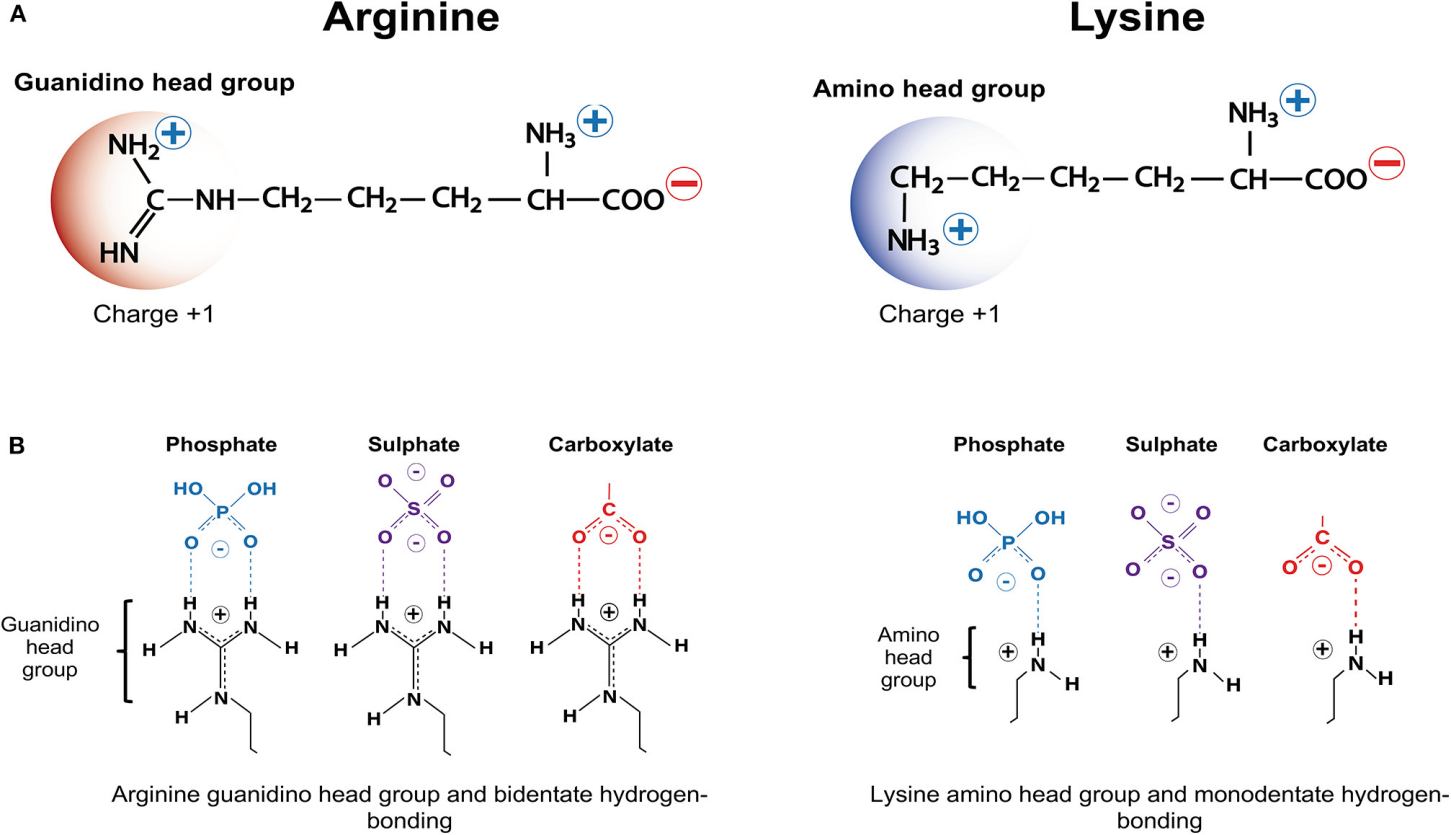
Positively charged amino acids arginine and lysine, and hydrogen bonding. **(A)** Arginine and lysine depicting positively charged guanindino head group and amino head group, respectively. **(B)** Arginine guanindino head groups and lysine amino head groups forming bidentate hydrogen-bonding and monodentate hydrogen-bonding, respectively, with phosphate, sulfate and carboxylate anionic moieties.

As the name suggests, critical factors for CARP neuroprotection are their positive charge and arginine content as well as, the ability to traverse membrane lipid bilayers. Whereas, cationic charge can be imparted by the presence of the positively charged amino acids arginine and lysine (Figure 1A), which have a net charge of +1 at pH 7, arginine is the amino acid essential for neuroprotection. Histidine, the other positively charged amino acid only provides a modest contribution to peptide charge with a net charge of +0.1 at pH 7. Furthermore, CARPs represent a broader class of bioactive peptides with a number of other properties that may contribute to their neuroprotective actions, including the ability to reduce intracellular calcium influx, antagonize cell surface receptor function, target mitochondria, scavenge reactive molecules, induce cell signaling, stabilize proteins, inhibit proteolytic enzymes, and reduce inflammation, and in addition to being neuroprotective also have anti-nociceptive, cardioprotective, anti-microbial and anti-cancer properties.

CARPs have demonstrated neuroprotection in *in vitro* neuronal injury models (e.g., excitotoxicity, oxygen-glucose deprivation), in *in vivo* models of acute central nervous system (CNS) injury (e.g., stroke, traumatic brain injury, perinatal hypoxia-ischemia, traumatic brain injury, spinal cord injury, and epilepsy) and in models of chronic neurodegenerative disorders (e.g., Parkinson's and Alzheimer's disease) and neuropathic pain (Tables 1–3). Furthermore, it is important to acknowledge that neuroprotective CARPs can be categorized into three main groups; (i) poly-arginine peptides, cationic arginine-rich cell-penetrating peptides (CCPPs) or peptides derived from proteins (Table 1); (ii) putative neuroprotective peptides fused to CCPPs (Table 2); and (iii) endogenous peptides (Table 3).

**Table 1:** CARPs with neuroprotective and other neuroactive properties.

**Peptide name**	**Peptide sequence**	**% Arginine**	**Net charge at pH 7**	**Neuronal injury model**	**References**
R6 and CARP 6-mers	RRRRRR-NH_2_, RRRRWW-NH_2_, rrrrrw-NH_2_, rrrrww-NH_2_, Ac-MCRRKR-NH_2_, Ac-LCRRKF-NH_2_, Ac-RRWWIR-NH_2_	33–100%	+4 to +6	Excitotoxicity, pain	(1, 2)
SS-31, SS-20	rDmtKF-NH_2_, FrFK-NH_2_	25%	+3	Stroke, MPTP, SCI, AD, pain	(3–7)
TAT, TAT-D	YGRKKRRQRRRG, ygrkkrrqrrrg	50%	+8	Excitotoxicity, stroke	(8–13)
Penetratin	RQIKIWFQNRRMKWKK	19%	+7	Excitotoxicity	(12)
R7, C-R5, C-R7, C-r7	RRRRRRR-NH_2_, C-s-s-CRRRRR-NH_2_, C-s-s-CRRRRRRR-NH_2_, C-s-s-crrrrr-NH_2_	71–100%	+6 to +8	Excitotoxicity	(14)
R8 to R15, R9D, R18, R18D, R22	RRRRRRRR to RRRRRRRRRRRRRRR, rrrrrrrrr-NH_2_, RRRRRRRRRRRRRRRRRR, rrrrrrrrrrrrrrrrrr, RRRRRRRRRRRRRRRRRRRRRR	100%	+6 to +22	Excitotoxicity, stroke, HIE, TBI, AD	(12, 15–27)
BEN2540, BEN0540, BEN1079	Ac-WGCCGRSSRRRRTR-NH_2_, Ac-PFLKRVPACLRLRR-NH_2_, Ac-RCGRASRCRVRWMRRRRI-NH_2_	29–44%	+4.9 to +8.9	Excitotoxicity	(15)
XIP, R9/X7/R9, NCXBP3	RRLLFYKYVYKRYRAGKQRG, RRRRRRRRRPGRVVGGRRRRRRRRR, RRERRRRSCAGCSRARGSCRSCRR-NH_2_	25–80%	+8 to +19	Excitotoxicity	(15)
LMWP	VSRRRRRRGGRRRR	71%	+10	Excitotoxicity	(16)
R10W4D, R10W8, R12W8a, R12F8, R12Y8	wwrrrrrwwrrrrr-NH_2_, WWRRRWWRRRRWWRRRWW, WWRRRRWWRRRRWWRRRRWW, FFRRRRFFRRRRFFRRRRFF, YYRRRRYYRRRRYYRRRRYY	55–71%	+11 to +12	Excitotoxicity	(16)
D3, D3D3, RD2	rprtrlhthrnr-NH_2_, rprtrlhthrnrrprtrlhthrnr-NH_2_, ptlhthnrrrrr-NH_2_	42%	+6.2 to +11.4	AD	(28–30)
IDR-1018	VRLIVAVRIWRR-NH_2_	33%	+5	HIE	(31)
Hi1a	NECIRKWLSCVDRKNDCCEGLECYKRRHSFEVCVPIPGFCLVKWKQC DGRERDCCAGLECWKRSGNKSSVCAPIT	9%	+3.3	Stroke	(32)
APP96-110	Ac-NWCKRGRQCKTHPH-NH_2_	14%	+4	TBI	(33–35)
COG133	Ac-LRVRLASHLRKLRKRLL-NH_2_	29%	+7.1	Excitotoxicity, HIE, TBI, EAE, LPS, AD	(36–41)
COG1410	Ac-ASAibLRKLAibKRLL-NH_2_	17%	+4	Stroke, SAH, TBI, ICH, SCI	(24, 42–52)
CN-105	Ac-VSRRR-NH_2_	60%	+3	Stroke, TBI, ICH	(53–55)
PRARIY	PRARIY	33%	+2	Stroke, SCI	(56, 57)
Syn 1020	Ac-RY(3-Cl)YRWR-NH_2_	50%	+3	Pain	(58)

*At the N-terminus, Ac indicates acetyl and at the C-terminus NH_2_ indicates amide. Lower case single letter code indicates D-isoform of the amino acid. Aib, 2-Aminoisobutyric acid or 2-methylalanine; s-s, disulfide bond; Y(3-Cl), 3-chloro-L-tyrosine. AD, Alzheimer's disease; CARPs, cationic arginine-rich peptides; Dmt, 2′,6′-dimethyl-L-tyrosine; EAE, Experimental autoimmune encephalomyelitis; HIE, hypoxia-ischaemia encephalopathy; ICH, intracerebral hemorrhage; LMWP, Low molecular weight protamine; LPS, Lipopolysaccharide; MPTP, 1-methyl-4-phenyl-1,2,3,6-tetrahydropyridine; MS, multiple sclerosis; PNI, peripheral nerve injury; SAH, subarachnoid hemorrhage; SCI, spinal cord injury; stroke, ischaemic stroke; TBI, traumatic brain injury*.

**Table 2:** Studies demonstrating neuroprotective and other neuroactive properties of peptides fused to TAT and other cell penetrating peptides.

**Peptide name**	**Peptide sequence**	**% Arginine**	**Net charge at pH 7**	**Neuronal injury model**	**References**
TAT-NR2B9c (NA-1)	YGRKKRRQRRR-KLSSIESDV	30%	+7	Excitotoxicity, stroke, HIE, ICH, AD, epilepsy, pain	(59–68)
JNKI-1D-TAT, JNKI-1-TAT	dqsrpvqpflnlttprkprpp-rrrqrrkkrg-NH_2_, GRKKRRQRRR-PP-RPKRPTTLNLFPQVPRSQD-NH_2_	29%	+12	Excitotoxicity, stroke, HIE, ICH, TBI, AD, SCI, SMA, epilepsy, pain	(60, 69–83)
TAT-JIP-1	GRKKRRQRRR-RPKRPTTLNLF	38%	+11	Excitotoxicity, stroke, GCI, PD	(84–86)
δSV1-1-TAT	YGRKKRRQRRR-SFNSYELGSL	28%	+7	Stroke	(87, 88)
TAT-JBD	GRKKRRQRRR-PP-RPKRPTTLNLFPQVPRSQDT	28%	+11	HIE, GCI	(89, 90)
TAT-NPEG4-(IETDV)2	YGRKKRRQRRR-(Peg)4-(IESDV)2	28%	+9	Stroke, pain, epilepsy, cortical spreading depression	(91–95)
JNK3-N-TAT	YGRKKRRQRR-RCSEPTLDVKI	29%	+6.9	PD	(96, 97)
Src40–49Tat	KPASADGHRGY-GRKKRRQRRR	33%	+9.1	Pain	(98)
TAT-Sab_KIM1_	GFESLSVPSPLDLSGPRVVAPP-RRRQRRKKRG-NH_2_	22%	+8	PD	(99)
TAT-CBD3	YGRKKRRQRRR-ARSRLAELRGVPRGL	38%	+11	Excitotoxicity, stroke, TBI, pain	(100–105)
R9-CBD3	RRRRRRRRR-ARSRLAELRGVPRGL	54%	+12		
TAT-CBD3A6K	YGRKKRRQRRR-ARSRLKELRGVPRGL	38%	+12		
TAT-CRMP-2	YGRKKRRQRR-GVPRGLYDGVCEV	26%	+6.9	Excitotoxicity, stroke, OGD	(106–108)
TAT-NR2B_ct_	YGRKKRRQRRR-KKNRNKLRRQHSY	37%	+14.1	Excitotoxicity, stroke	(109–111)
TAT-NR2B_ct_s	YGRKKRRQRRR-NRRRNSKLQHKKY	35%	+14.1	Excitotoxicity	(109, 110)
Tat-D2_LIL3−29−2_	YGRKKRRQRRR-MKSNGSFPVNRRRMD	34%	+11	Depression	(112)
Penetratin-COG133 (COG112)	Ac-RQIKIWFQNRRMKWKK-LRVRLASHLRKLRKRLL-NH_2_	24%	+14.1	TBI, EAE, AD, axonal regeneration, spinal cord demyelination	(40, 41, 47, 113–115)
TAT-NR2Bct-CTM	YGRKKRRQRRR-KKNRNKLRRQHSY-KFERQKILDQRFFE	35%	+15.1	Stroke	(116)
CN2097	RRRRRRRC-s-s-CKNYKKTEV (cyclic or linear)	41%	+9	Excitotoxicity, pain	(14, 117)
P42-TAT	AASSGVSTPGSAGHDIITEQPRS-GG-YGRKKRRQRRR	19%	+7.1	Huntington's disease	(118)
TAT-p53DM	YGRKKRRQRRR-RVCACPGRDRRT	43%	+11	288,289	(14, 109, 119, 120)
TAT-p53DMs	YGRKKRRQRRR-CCPGECVRTRRR	43%	+11	Excitotoxicity	(109)
TAT-CN21	YGRKKRRQRR-KRPPKLGQIGRSKRVVIEDDR	29%	+11	Excitotoxicity, stroke, GCI	(121–123)
PYC36-TAT, PYC36D-TAT	GRKKRRQRRRGG-LQGRRRQGYQSIKP, pkisqygqrrrgqlgg-rrrqrrkkrg	35%	+12	Excitotoxicity	(10)
TAT-GluR6-9c	YGRKKRRQRR-RLPGKETMA	32%	+8	Excitotoxicity, GCI, stroke, OGD	(124–126)
TAT-mGluR1	YGRKKRRQRRR-VIKPLTKSYQGSGK	24%	+11	Excitotoxicity, HIE, SAH	(127–129)
TAT-K13	YGRKKRRQRR-KEIVSRNKRRYQED	33%	+9	Stroke	(130)
TAT-Indip	YGRKKRRQRRR-GEPHKFKREW	33%	+9.1	Excitotoxicity, ALS	(109, 131)
TAT-Indip-K/R	YGRKKRRQRRR-GEPHRFRREW	43%	+9.1	Excitotoxicity	(109)
TAT-GESV, D-TAT-GESV	RRRQRRKKRG-YAGQWGESV, rrrqrrkkrg-yagqwgesv	32%	+7	Excitotoxicity, HIE, pain	(132–134)
TAT-NEP1-40	YGRKKRRQRRR-RIYKGVIQAIQKSDEGHPFRAYLESEV AISEELVQKYSNS	16%	+7.1	Stroke, OGD	(135, 136)
TAT-NBD	YGRKKRRQRRR-TALDWSLWQTE	27%	+6	HIE	(137)
TAT-ψεHSP90	YGRKKRRQRRR-PKDNEER	39%	+8	Stroke, OGD	(138)
TAT-Bec	YGRKKRRQRRR-GG-TNVFNATFEIWHDGEFGT	19%	+6.1	SCI	(139)
TAT-gp91ds	GRKKRRQRRR-CSTRIRRQL-NH_2_	47%	+12	SCI, TBI, SAH	(140–142)
TAT-ISP	GRKKRRQRRR-CDMAEHMERLKANDSLKLSQEYESI-NH_2_	20%	+6	SCI	(143)
Tat-Cav3.2-III-IV	YGRKKRRQRRR-EARRREEKRLRRLERRRRKAQ	50%	+16	Pain	(144)
TAT-μCL	YGRKKRRQRRR-PPQPDALKSRTLR	33%	+10	Retinal degeneration	(145)
ST2-104	RRRRRRRRR-ARSRLAELRGVPRGL	54%	+12	Pain	(146)
TAT-STEP	YGRKKRRQRRR -GLQERRGSNVSLTLDM	30%	+8	Excitotoxicity, stroke, OGD	(147)
TAT-K	YGRKKRRQRRR-PP-LNRTPSTVTLNNNT	26%	+9	Excitotoxicity	(148)
TAT-P110	YGRKKRRQRRR-GG-DLLPRGT	35%	+9	Stroke, Huntington's disease	(149, 150)
TAT-C6	GRKKRRQRRR-CRRGGSLKAAPGAGTRR	37%	+14	Stroke	(151)
Analog 4 and 5	Y-βP-WFGG-RRRRR, YaWFGG-RRRRR	45%	+5	Pain	(152)
Aβ1-6_A2V_TAT(D)	grkkrrqrrr-gggg-dvefrh	35%	+8.1	AD	(153)
DEETGE-CAL-TAT	RKKRRQRRR-PLFAER-LDEETGEFLP-NH_2_	28%	+5	GCI	(154)
TAT-T406	RKKRRQRR-IAYSSSETPNRHDML	29%	+7.1	Pain	(155)
TAT-21-40	RKKRRQRRR-RIPLSKREGIKWQRPRFTRQ	38%	+14	Excitotoxicity, stroke, OGD	(156)
TAT-C1aB	YGRKKRRQRRR-HLSPNKWKW	30%	+10.1	Excitotoxicity, stroke	(157)
TAT-2ASCV	YGRKKRRQRRR-TVNEKVSC	31%	+8	Pain	(158)
TAT-NTS	YGRKKRRQRRR-RSFPHLRRVF-NH_2_	43%	+12.1	Stroke, OGD	(159)
TAT-CBD3M5L	YGRKKRRQRR-ARSRMA	44%	+9	Pain	(160)
TDP-r8	YrFG-rrrrrrrr-G	69%	+9	Pain	(161)
TAT-Pro-ADAM10	YGRKKRRQRR-PKLPPPKPLPGTLKRRRPPQP	27%	+14	Huntington's disease	(162)

*At the N-terminus, Ac indicates acetyl and at the C-terminus NH_2_ indicates amide. Lower case single letter code indicates D-isoform of the amino acid. Aib, 2-Aminoisobutyric acid or 2-methylalanine; s-s, disulfide bond; AD, Alzheimer's disease; ALS, amyotrophic lateral sclerosis; EAE, Experimental autoimmune encephalomyelitis; GCI, global cerebral ischaemia; HIE, hypoxia-ischaemia encephalopathy; ICH, intracerebral hemorrhage; OGD, oxygen glucose deprivation; PD, Parkinson's disease; SAH, subarachnoid hemorrhage; SCI, spinal cord injury; SMA, spinal muscular atrophy; stroke, ischaemic stroke; TBI, traumatic brain injury*.

**Table 3:** Endogenous CARPs with neuroprotective and cytoprotective properties.

**Peptide name**	**Peptide sequence**	**% Arginine**	**Net charge at pH 7**	**Neural/cell injury model**	**References**
Apelin-13	QRPRLSHKGPMPF	15%	+3.1	Excitotoxicity, stroke, TBI, ICH, SCI, pain	(163–176)
Apelin-17	KFRRQRPRLSHKGPMPF	23%	+6.1		
Apelin-36	LVQPRGSRNGPGPWQGGRRKFRRQRPRLSHKGPMPF	20%	+10.1		
Dynorphin A 1-13,	YGGFLRRIRPKLK, YGGFLRRIRPKLKWDNQ	23%	+5	Pain, stroke, LPS	(177–179)
Dynorphin A 1-17					
PACAP38	HSDGIFTDSYSRYRKQMAVKKYLAAVLGKRYKQRVKNK	11%	+9.1	Excitotoxicity, stroke, GCI, TBI, PD, pain	(180–185)
Ghrelin	GSSFLSPEHQRVQQRKESKKPPAKLQPR	11%	+5.1	Stroke, PD, AD, SAH, epilepsy, TBI, pain	(186–192)
Humanin	MAPRGFSCLLLLTSEIDLPVKRRA	12%	+2	Excitotoxicity, stroke, AD, SAH, HIE	(193–197)
PR-39 PR-11	RRRPRPPYLPRPRPPPFFPPRLPPRIPPGFPPRFPPRFP RRRPRPPYLPR	25% 45+	+10 +5	Hypoxia, ischaemia/reperfusion, oxidative stress: endothelial cells, HeLa cells, myocardial infarction	(198–200)
Protamine	PRRRRSSSRPVRRRRRPRVSRRRRRRGGRRR	66%	+21	Excitotoxicity, stroke	(16)

*AD, Alzheimer's disease; GCI, global cerebral ischaemia; HIE, hypoxia-ischaemia encephalopathy; ICH, intracerebral hemorrhage; LPS, Lipopolysaccharide; SAH, subarachnoid hemorrhage; SCI, spinal cord injury; PD, Parkinson's disease; stroke, ischaemic stroke; TBI, traumatic brain injury*.

The aim of this review is to highlight the recognition of CARPs as a novel class of peptide with great promise for the treatment of acute and chronic neurological disorders, and in so doing summarize their known neuroprotective mechanisms of action, as well as other potential actions whereby they may exert beneficial effects in injured or affected cells. Within this group of compounds are included many putative neuroprotective peptides fused to CCPPs (e.g., TAT, R9, penetratin) that have been developed (Table 2). In this review, such peptides are also classified as CARPs, and we propose that in many, if not all instances their putative neuroprotective effects may actually be mediated by the arginine content and positive charge of the carrier and/or cargo peptide, rather than the cargo peptide itself.

## Generic Features of Neuroprotective CARPs

In general terms, neuroprotective CARPs typically possess the following properties: (i) range in size from 4 to 40 amino acids; (ii) positive net charge ≥ +2 to +20; (iii) one or more positively charged arginine residues that comprise between 20 and 100% of the peptide; (iv) other positively charged amino acids namely lysine and histidine; (v) amphiphilicity due to the presence of both hydrophilic (e.g., arginine, lysine) and hydrophobic (e.g., tryptophan, phenylalanine, tyrosine) amino acids; and (vi) endocytic and/or non-endocytic cell membrane traversing properties, including the ability to cross the blood-brain and blood-spinal cord barriers (BBB/BSCB). Invariably, CARPs are commercially or chemically synthesized using solid-phase peptide synthesis. One exception is the CARP, protamine (Table 3), which is purified from salmon milt or generated recombinantly. Due to the capacity of CARPs to traverse cellular membranes and localize to different organs within the body, they have been the subject of several experimental and review articles examining their bioavailability (201–203) and therefore this subject is not covered in this review.

## Historical Overview of CARPs and Neuroprotection Studies

**Figure 2:**
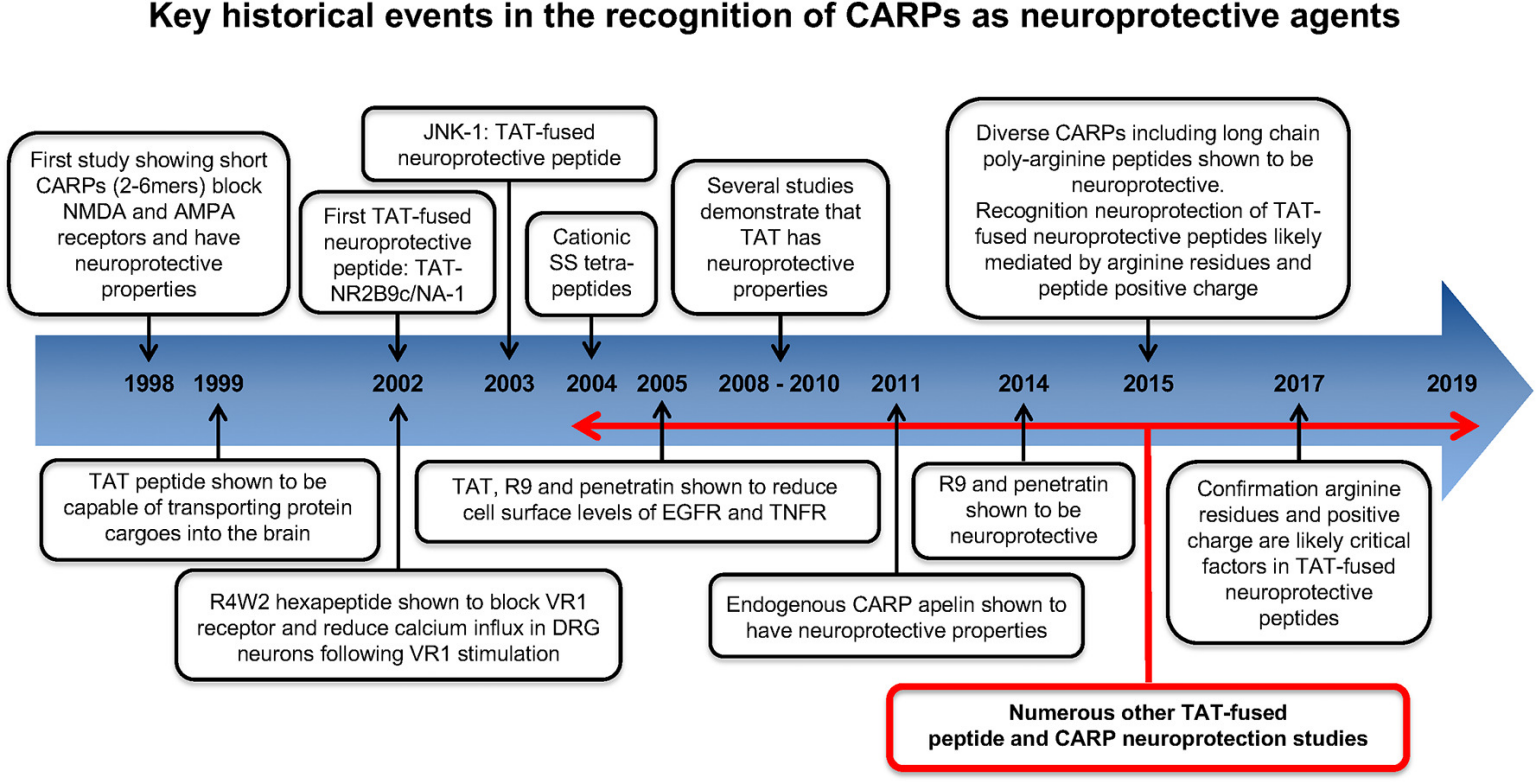
Historical time-line for the recognition of CARPs as neuroprotective agents.

Key historical events in the recognition and application of CARPs as neuroprotective agents are summarized in Figure 2. The first study to identify the neuroprotective properties of CARPs was in 1998 when Ferrer-Montiel et al. (1) screened a 6-mer peptide library containing over 49,000 different peptides for their ability to block glutamate-evoked ionic currents in *Xenopus* oocytes expressing the NR1 and NR2A NMDA receptor subunits. Hexapeptides containing at least two arginine (R) residues at any position as well as one or more lysine (K), tryptophan (W), and cysteine (C) residues displayed ionic current blocking activity. Further analysis revealed that C-carboxyl amidated (-NH_2_; note C-carboxyl amidation removes the negatively charged COO^−^ C-terminus thereby increasing peptide net charge by +1) dipeptides RR-NH_2_ (net charge +3) and RW-NH_2_ (net charge +2) were also capable of blocking NMDA receptor activity. Similarly, certain amino acid residues within arginine-rich hexapeptides inhibited the NMDA receptor blocking ability of the peptide (e.g., RFMRNR-NH_2_; net charge +4, was ineffective; M, methionine; N, asparagine). In addition, increasing oligo-arginine peptide length from 2 to 6 resides (e.g., R2-NH_2_ vs. R3-NH_2_ vs. R6-NH_2_) increased blocking activity. In a NMDA excitotoxicity model (NMDA: 200 μM/20 min) using cultured hippocampal neurons, arginine-rich hexapeptides (Table 1), especially those also containing one or two tryptophan residues displayed high-levels of neuroprotection, and the neuroprotective action of the peptides was not stereo-selective with L- and D-isoform peptides showing similar efficacy. The ability of tryptophan to improve peptide neuroprotective efficacy is of particular interest as tryptophan residues also increase the uptake efficacy of CCPPs (204–208).

Other observations concluded that: (i) whereas cationic arginine-rich hexapeptides were highly efficient at blocking NMDA receptor evoked ionic currents (80–100%), some peptides (e.g., RRRCWW-NH_2_ and RYYRRW-NH_2_) also blocked AMPA receptor currents by over 60%. In subsequent studies, the peptide RRRRWW-NH_2_ was demonstrated to antagonize the vanilloid receptor 1 (VR1; also known as the transient receptor potential cation channel subfamily V member 1; TRPV1) mediated currents in a *Xenopus* expression system and reduce calcium influx in rat dorsal root ganglion neurons following capsaicin or resiniferatoxin VR1 receptor stimulation (2, 209).

### Neuroprotective Properties of Cationic Arginine-Rich Cell-Penetrating Peptides TAT, R9, and Penetratin

**Figure 3:**
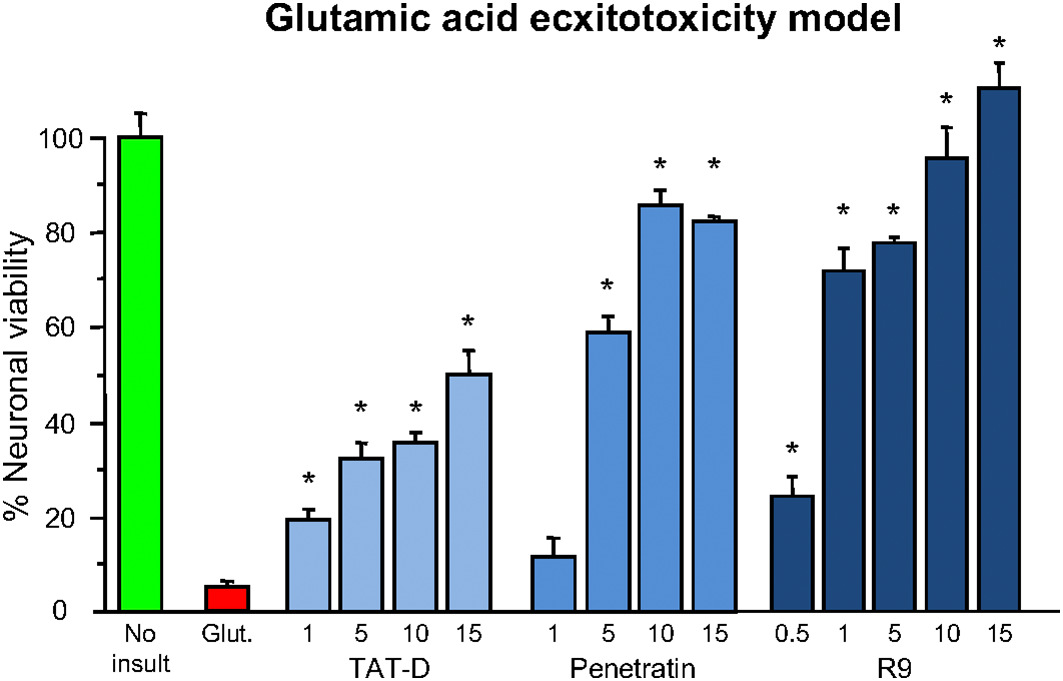
Neuroprotective efficacy of cationic arginine-rich cell-penetrating peptides in glutamic acid excitotoxicity model. Peptides present in neuronal cultures for 10 min before and during (half concentration) 5-min glutamic acid exposure. Neuronal viability measured 24 h following glutamic acid exposure. Concentration of peptide in μM. MTS assay data were expressed as percentage neuronal viability with no insult control taken as 100% viability and glutamic acid control (Glut.) taken as 5% (mean ± SE; *n* = 4; ^*^P < 0.05). Adapted from Meloni et al. (12).

Shortly after the work of Ferrer-Montiel et al. (1), the CCPP TAT (see Table 1 for sequence and net charge) was demonstrated to have the capacity to transport large protein cargos across the BBB (210). Subsequently, the TAT peptide became increasingly utilized as a carrier molecule to deliver various cargos into the brain, including putative neuroprotective peptides and proteins (Figure 2). To date over fifty different TAT-fused neuroprotective peptides have been shown to have positive effects in different *in vitro* and/or animal CNS injury models (Table 2). However, not surprisingly in light of the Ferrer-Montiel et al. (1) findings, experiments in other laboratories demonstrated that the TAT peptide itself possesses modest neuroprotective actions in *in vitro* excitotoxicity and *in vivo* ischemic injury models (8–11). Subsequently, it was reported that the CCPPs, R9 (Table 1), and penetratin (Table 1) were 17- and 4.6-fold, respectively more neuroprotective than TAT in a severe cortical neuronal glutamic acid excitotoxicity cell death model (glutamic acid: 100 μM/5 min; Figure 3) (12). These findings also raised the first clues that the neuroprotective actions of putative neuroprotective peptides fused to CCPPs may in fact be mediated by the carrier peptide.

### Further Validation and Characterization of CARPs as Neuroprotective Agents

Later, *in vitro* studies confirmed that other CARPs (e.g., protamine, LMWP, XIP; Tables 1, 3) and long-chain poly-arginine peptides (Table 1) were also highly neuroprotective, with efficacy increasing with increasing arginine content and peptide positive charge, plateauing at around 15–18 arginine residues for arginine polymers (15, 16). Furthermore, the requirement for arginine residues, rather than lysine residues, was demonstrated to be critical for neuroprotection, with the K10 peptide (10-mer of lysine; net charge +10) displaying limited efficacy in a neuronal glutamic acid excitotoxic model (15). In addition, the importance of peptide charge was confirmed by the finding that the glutamic acid containing neutrally charged R9/E9 peptide (RRRRRRRRREEEEEEEEE; net charge 0; E = glutamic acid) displayed no neuroprotection in the excitotoxic model (15).

Based on the above findings, it was hypothesized that CARP neuroprotection is largely mediated by the positively charged guanidinium head-group, which is unique to arginine (Figure 1A) (note: lysine possesses a positively charged amide group; Figure 1A) (15, 109). These findings also support the notion that peptide neuroprotective efficacy appears to be correlated with the same features that are critical for the endocytic and/or non-endocytic membrane traversing properties of CCPPs (14, 15, 109, 211). It was also demonstrated in an *in vitro* glutamic acid excitotoxicity model that the hydrophobic aromatic amino acids tryptophan, and to a lesser extent phenylalanine and tyrosine can significantly improve CARP neuroprotective efficacy. In contrast, alanine and glycine resides reduce peptide neuroprotective efficacy (15, 16). Importantly, tryptophan residues are also known to increase the cell-penetrating properties of CCPPs, providing further evidence that neuroprotection is closely linked to the peptide membrane traversing capacity of the peptides.

Studies have also demonstrated that a 10-min pre-treatment of neuronal cultures with CARPs induces a pre-conditioning neuroprotective response lasting up to 2–5 h post-treatment (15–17). Similar to the findings of Ferrer-Montiel et al. (1), it was also observed that there was no stereo-selectivity in terms of neuroprotective efficacy of L- and D-enantiomer CARPs, which suggests that with respect to neuroprotection, peptide electrostatic interactions are more important than peptide structural interactions of the peptide with specific biological targets. Importantly, CARPs have the capacity to significantly inhibit neuronal intracellular calcium influx in the glutamic acid excitotoxicity model (15–17, 109).

Consistent with *in vitro* findings, CARPs (e.g., R9D, R12, R18, R18D, protamine; Tables 1, 3) were also demonstrated to provide significant neuroprotection and improve functional outcomes in rat models of permanent and/or transient middle cerebral artery occlusion (MCAO), perinatal hypoxia-ischemia and traumatic brain injury (15, 16, 18–24, 212) and a non-human primate MCAO stroke model (26). Positive neuroprotective effects with R9D and R18D, which are the D-enantiomers of R9 and R18, also confirmed the lack of stereo-specificity for CARP efficacy *in vivo*.

In 2015, Marshall et al. (14) also confirmed the *in vivo* neuroprotective properties of CARPs including poly-arginine R7 (Table 1), as well as the TAT and TAT-NR2B9c (also known as NA-1; Table 2) peptides in rat retinal ganglion cells exposed to NMDA (20 nmol; 3 μL intravitreal injection). The study also demonstrated that CARPs containing a terminal cysteine residue improved neuroprotective efficacy; this could be due to the cysteine residue improving peptide stability and/or enhancing anti-oxidant properties. Marshall et al. (14) also considered that it was likely that the cell-penetrating properties of the CARPs along with the guanidinium head group of arginine and peptide positive charge were the “driving force” for neuroprotection. Furthermore, and as proposed by Meloni et al. (211), Marshall et al. (14) also suggested that cargo peptides designed to inhibit cell death following NMDA excitotoxicity (e.g., peptides CN2097; CKNYKKTEV and NR2B9c; KLSSIESDV) and fused to a CCPP (e.g., R7 for CN2097 and TAT for NR2B9c) were unlikely to be the active component mediating neuroprotection in the retinal ganglion cell NMDA excitotoxic injury model.

In 2017, McQueen et al. (110) re-evaluated the neuroprotective mechanism of action of the death-associated protein kinase 1 protein (DAPK1) blocking peptide TAT-NR2Bct (Table 2) and its scrambled control TAT-NR2Bcts (Table 2). DAPK1 is a calcium-calmodulin regulated protein activated in neurons following NMDA receptor over-stimulation as occurs in ischemia mediated excitotoxicity. TAT-NR2Bct was designed to competitively inhibit activated DAPK1 binding to the NR2B subunit protein, and thereby block subsequent downstream damaging cellular events caused by NMDA receptor over-activation. Interestingly, Meloni et al. (109) had earlier examined the TAT-NR2Bct and TAT-NR2Bcts peptides and demonstrated high neuroprotective efficacy for both peptides in the glutamic acid excitotoxicity model. Therefore, it was not surprising that McQueen et al. (110) also found that both TAT-NR2Bct and TAT-NR2Bcts, along with a randomly designed CARP (RRRTQNRRNRRTSRQNRRRSRRRR; net charge +15) were neuroprotective in a neuronal NMDA excitotoxicity model. On the basis of their findings they concluded that neuroprotection was dependent on peptide positive charge and independent of peptide sequence and DAPK1 signaling.

Taken together, the above studies provide irrefutable evidence of the neuroprotective properties of CARPs in various experimental situations and in doing so, raise two important issues in regard their application in neuroprotection: (i) what are the precise neuroprotective mechanisms operating; and (ii) the need to re-evaluate studies using CARPs and CCPPs for the delivery of neuroactive cargos into the CNS, particularly putative neuroprotective peptides. Both these topics are discussed below. Also, because it is likely that CARPs interact with negatively charged cell membrane structures, an interaction that appears to be critical for neuroprotection, the mechanisms associated with the affinity of CARPs to cell membranes will also be discussed. Interestingly, it is the interaction of CARPs with negatively charged bacterial and cancer cell cytoplasmic membrane structures that is considered to be one of the mechanisms responsible for their anti-bacterial and anti-cancer properties (213, 214).

## Putative Neuroactive Peptides Fused to Cationic Arginine-Rich Cell-Penetrating Peptides and Neuroprotection

Given that CARPs possess intrinsic neuroprotective properties raises questions regarding the mode of action of other putative neuroprotective peptides when they are fused to a carrier CCPP (Table 2). As alluded to above, it is likely that the neuroprotection provided by such putative neuroprotective peptides fused to CCPPs, is mediated not by the actions of the cargo molecule *per se*, but by the carrier itself with potency being further enhanced by the amino acid content (e.g., arginine, lysine, cysteine, and tryptophan resides) and/or stability provided by the cargo peptide. In essence, a putative neuroprotective peptide fused to an arginine-rich cell-penetrating carrier peptide will possess the properties of a CARP; the only exception being if a negatively charged cargo peptide neutralizes the positive charge of the carrier peptide.

In 2015 we published a review article (109) highlighting the likelihood of the neuroprotective mechanism of action of putative neuroprotective peptide fused to cell-penetrating carrier peptides being mediated by the carrier molecule. Three of the most commonly used TAT-fused neuroprotective peptides TAT-NR2B9c, TAT-JNKI-1 and TAT-CBD3, as well as several other less characterized TAT-fused peptides (e.g., TAT-p53DM, TAT-s-p53DM, TAT-NR2Bct, TAT-NR2Bcts, Indip/IndipK-R) were analyzed based on theoretical grounds, and on our own and other previous experimental studies in relation to neuroprotective mechanism of action. Following this analysis, we provided several lines of evidence to support the view that TAT-fused neuroprotective peptides are behaving as neuroprotective CARPs, and not by the proposed intended mechanism of action of the cargo peptide. This evidence included: (1) the ability of the peptides to reduce intracellular calcium influx, even though this was never an intended mechanism of action of the cargo peptide; (2) despite targeting intracellular proteins, the peptides often reduced surface expression or interfered with plasma membrane ion channel receptors; (3) lack of efficacy and inability of the peptide to reduce neuronal calcium influx when introduced directly into the cell; (4) improved peptide efficacy when TAT was replaced with R9 (increasing peptide positive charge and arginine content) or replacing neutral or negatively charged amino acids with positively charged arginine or lysine; (5) decreased peptide efficacy when replacing amino acids with alanine, which is known to reduce membrane traversing properties of cell-penetrating peptides; (6) demonstrating neuroprotective properties of CCPP-fused scrambled cargo control peptides; and (7) due to endosomal entrapment and/or peptide degradation it is possible cargo peptides have a limited capacity to interact with their intended intracellular target. Importantly, the subsequent studies of Marshall et al. (14) and McQueen et al. (110) (described above) further validate the view that the mechanism of action of TAT-fused neuroprotective peptides is likely to be mediated by the carrier peptide, and by extension the arginine content and positive charge of the peptide.

In order to confirm the specific action of a neuroactive peptide cargo fused to a carrier CCPP, we recommend that the neuroprotective or other intended neuroactive actions of the peptide should be reassessed after the introduction of arginine substitutions into the cargo peptide. The introduction of arginine residues into the cargo peptide should abolish the proposed/intended neuroprotective action of the cargo peptide. However, if the action of the carrier-cargo peptide is maintained or enhanced it is likely that the neuroprotective action of the peptide was mediated by the cationic and arginine-rich properties of the peptide. Alternatively, the peptide could be synthesized in the same amino acid sequence (as opposed to retro-inversely) with D-isoform amino acids, which would drastically alter the peptide's steric structure and binding specificity/affinity to its intended target, whereas its electro-physiochemical properties would be similar. Finally, the CCPP carrier molecule could be replaced with a non-arginine containing cell-penetrating peptide (e.g., TP10 or MAP).

## CARPs and Their Interaction With Cellular Membranes

CARPs have the capacity to form electrostatic interactions with anionic phosphate, sulfate and carboxylate moieties (Figure 1B) present on structures found in the plasma membrane and in membranes of cellular organelles (e.g., mitochondria, nucleus, endoplasmic reticulum, golgi, endosomes). These anionic chemical moieties are located within membrane proteoglycans (heparin sulfate proteoglycans: HSPGs; chondroitin sulfate proteoglycans: CSPGs; dermatan sulfate proteoglycans: DSPGs; keratin sulfate proteoglycans: KSPGs), glycoproteins, glycosphingolipids, and phospholipids as well as negatively charged aspartate and glutamate residues within protein receptors and other protein structures embedded in cellular membranes (Supplementary Table 1).

Negatively charged phosphate groups are a component of phospholipids that make-up cellular membrane bilayers (e.g., plasma membrane, inner and outer mitochondrial membrane, nuclear membrane, and endoplasmic reticulum membrane). There are at least five negatively charged membrane phospholipids including the mitochondrial membrane specific phospholipid cardiolipin, which possess a net charge of between −1 to −4 at pH 7 (Supplementary Table 1).

Proteoglycans a type of glycoprotein found on the surface of most cells and consist of a protein core and glycosaminoglycans (GAGs), which are long un-branched polysaccharides consisting of a repeating disaccharide subunit. Negatively charged sulfate groups are located on the polysaccharide repeating disaccharide subunits. In addition, the monosaccharide sialic acid is located at the end of the sugar chains attached to glycoproteins and has a negatively charged carboxyl group. Glycoproteins have important cellular functions, such as cell surface ligand receptor binding, cell signaling, cell adhesion, endocytosis, and binding extracellular matrix molecules (e.g., growth factors, enzymes, protease inhibitors, chemokines).

Glycolipids consist of a membrane lipid moiety covalently attached to a monosaccharide or polysaccharide. Glycolipids, namely glycosphingolipids, are negatively charged due to the presence of sialic acid. A glycosphingolipid containing one or more sialic acid residues is also known as a ganglioside. Gangliosides are expressed on most cells, but are more abundantly expressed on the cell surface of neurons, and are found ubiquitously throughout the CNS (215). They play a key role in modulating ion channel function, receptor signaling, cell-to-cell recognition and adhesion and regulation of neuronal excitability (216, 217). Membrane protein receptors rich in the acidic amino acids aspartate and glutamate also possess a negatively charged carboxylic moiety on their side chain.

With respect to the interaction of CARPs with anionic moieties, the positively charged arginine guanidinium head group forms bidentate hydrogen bonds with sulfates, carboxylates and phosphates, whereas the positively charged lysine amide head group forms weaker monodentate hydrogen bonds (Figure 1B). In addition, arginine and lysine cationic side chains can form salt bridges with the negatively charged aspartate and glutamate carboxylate C-termini, and cation-π interactions with the aromatic amino acids tryptophan, phenylalanine and tyrosine in proteins (218, 219). Interestingly, many neurotransmitters and drug-receptor interactions involve cation-π interactions (219). Together, the different electrostatic interaction between CARPs and plasma membrane structures can induce cellular uptake of the peptide by endocytic and non-endocytic pathways (220–224). Furthermore, peptide charge, arginine content and arginine distribution within the peptide, and the extent and density of the negatively charged moieties present on the cell surface play a significant role in terms of uptake efficacy (225–227). Peptide positive charge and arginine guanidinium head groups are also critical elements responsible for the ability of CARPs to target organelle membranes, such as the outer and inner mitochondrial membranes, which contain the negatively charged phospholipids cardiolipin (charge −2) and phosphatidylinositol 4, 5-bisphosphate (PIP2; charge −4) (Supplementary Table 1).

Importantly, studies have demonstrated that peptide characteristics that are known to increase the cell membrane traversing properties of CARPs, such as arginine content, peptide charge and presence of the aromatic amino acid tryptophan are also linked to increased peptide neuroprotective potency (1, 15, 16, 211).

## CARPs Have Multimodal Neuroprotective Mechanisms Of Action

**Figure 4:**
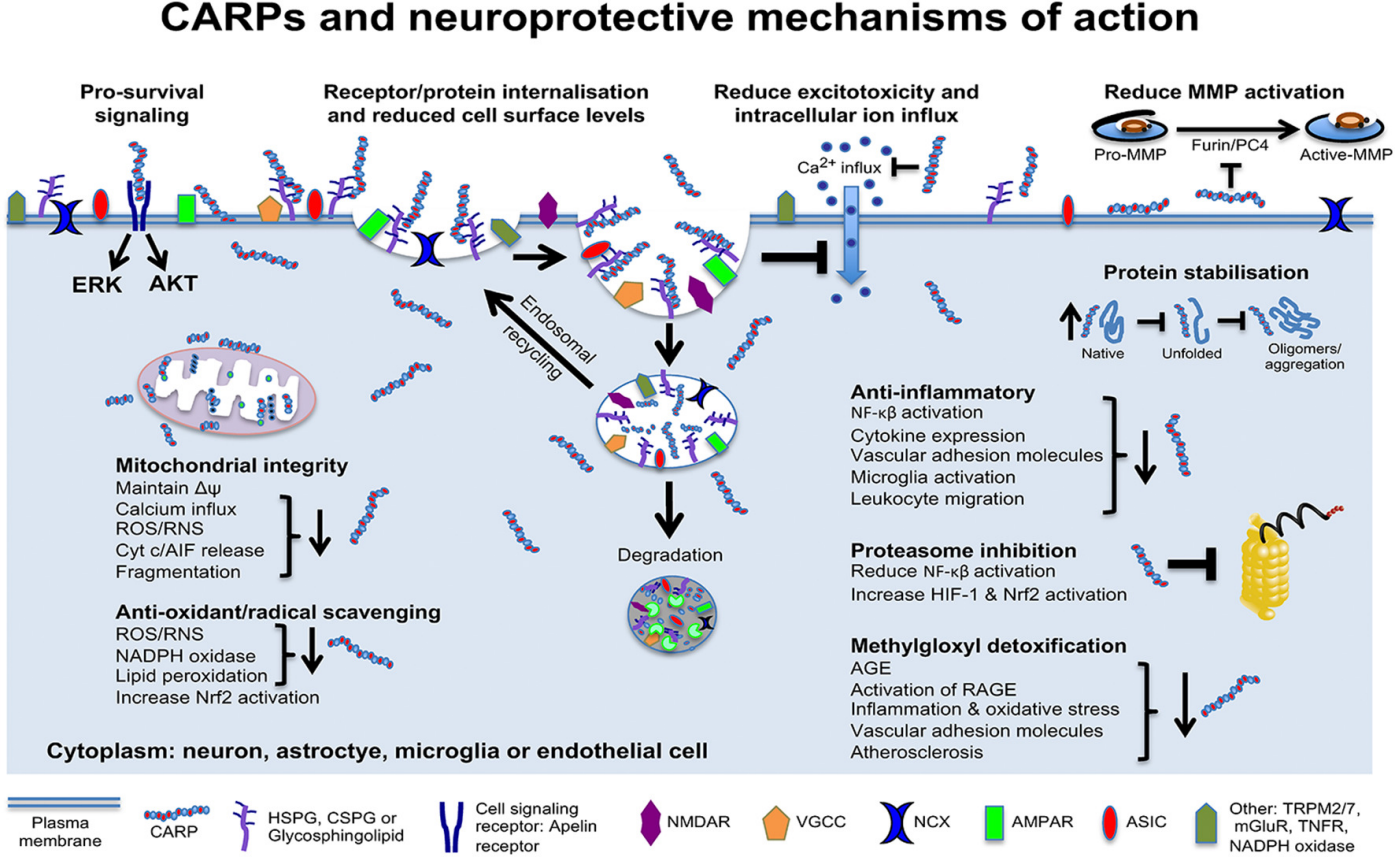
Schematic representation of CARP neuroprotective mechanisms of action. Model applies to neurons and potentially astrocytes, brain endothelial cells, oligodendrocytes, pericytes, and microglia. AGE, advanced glycation end products; RAGE, AGE receptors; AIF, apoptosis inducing factor; AKT, protein kinase B; Cyt c, cytochrome c; ERK, extracellular signal–regulated kinase; HIF-1, hypoxia-inducible factor-1; MMPs, matrix metalloproteinases; Δψ, mitochondrial transmembrane potential; NF-κB, nuclear factor kappa-light-chain-enhancer of activated B cells; Nrf2, nuclear factor erythroid 2-related factor 2; RNS, reactive nitrogen species; ROS, reactive oxygen species. NMDAR, N-methyl-D-aspartate receptor; AMPAR, α-amino-3-hydroxy-5-methyl-4-isoxazolepropionic acid receptor; NCX, sodium calcium exchanger; VGCC, voltage-gated calcium channels; ASIC, acid-sensing ion channels; TRPM2/7, transient receptor potential cation channels 2 and 7; mGluR, metabotropic glutamate receptor; TNFR, tumor necrosis factor receptor.

Data obtained in our laboratory and others using neuronal and non-neuronal cells indicate that CARPs have multimodal mechanisms of action targeting cell surface ion channel receptors and other receptors, mitochondria, proteolytic enzymes, oxidative stress/free radical molecules, protein stability, and pro-survival signaling, as well as having anti-inflammatory and immune regulatory actions (Figure 4). Evidence supporting these different neuroprotective mechanisms is provided below.

### Inhibition of Excitotoxic Neuronal Death and Excitotoxic Neuronal Calcium Influx

Our laboratory has established that CARPs are highly effective at reducing excitotoxic neuronal death and that they have the capacity to reduce glutamic acid induced neuronal calcium influx (9, 10, 15, 19, 109). These findings provide a mechanism in which CARPs inhibit glutamate-evoked ionic currents in *Xenopus* oocytes expressing NMDA receptors (1), and NMDA excitotoxic neuronal death in neuronal cultures *in vitro* and retinal ganglion cells *in vivo* (14, 59, 69, 110). In addition, other CARPs reduce potassium depolarization-induced calcium-influx (e.g., R9-CBD3-A6K, TAT-L1, TAT-ct-dis) and sodium currents and sodium influx (e.g., t-CSM) in cultured dorsal root ganglion neurons (see Supplementary Table 2 for details).

The ability of CARPs to reduce glutamate receptor and other receptor mediated intracellular neuronal calcium influx is likely to be a primary mechanism accounting for their neuroprotective efficacy in protecting neurons in injury models associated with excitotoxicity and excessive neuronal intracellular calcium influx. As a mechanism whereby CARPs act to reduce the intracellular influx of calcium and potentially other ions, we hypothesized (109) that CARPs have the capacity to induce the endocytic internalization of cell surface ion channel receptors (Figure 4). In support of this hypothesis we subsequently showed that R12, as well as the TAT-fused neuroprotective peptide TAT-NR2B9c, reduces neuronal cell surface expression of the glutamate receptor subunit protein, NR2B (228). Importantly, several CCPPs (e.g., TAT, penetratin, R9) have also been demonstrated to reduce TNF (tumor necrosis factor) and EGF (epidermal growth factor) receptors in non-neuronal cells via an endocytic internalization mechanism (Supplementary Table 2) (229).

### Interaction With Membrane Ion Receptors/Channels/Transporters

Many other studies have described the ability of CARPs to reduce neuronal and non-neuronal cell surface levels and/or activity of NMDA receptors, and other ion and non-ion channel receptors (see Supplementary Table 2). While these studies provide ample evidence for the ability of CARPs to perturb cell surface receptors it raises the question how different peptides with diverse amino acid sequences possess the ability to reduce cell surface levels and/or antagonize receptor function. As mentioned above, one mechanism involves CARP induced internalization of cell surface receptors. However, it is also possible CARPs antagonize ion channel receptor function by electrostatic interactions. For example, CARP electrostatic interactions with receptor anionic moieties may alter receptor function or interfere with ion transport within the receptor pore. In support of this, the guanidine moiety in arginine residues and in other molecules play a critical role in voltage-gated and ligand-gated ion channel function (see section Compounds Containing the Guanidinium Moiety and Neuroprotection) (230–237). For example, the guanidino moiety in agmatine, a molecule with neuroprotective properties (Supplementary Table 3), has been identified as being capable of interacting with a site within the NMDA receptor channel and calcium voltage channels and blocking their function (238). Interestingly, polyamines (e.g., putrescine and spermine) a class of compounds that also contain positively charged amino groups also have the capacity to block ion channels, including glutamate receptor and potassium channels (239). Given that positive charge is a critical factor for CARP neuroprotection and charge is independent of peptide amino acid sequence, provides additional support for a mechanism involving an electrostatic interaction perturbing ion channel function. Hence, there is good evidence to indicate that the structure and charge of the guanidine moieties in CARPs have the capacity to block ion channel receptor function, providing an additional mechanism whereby the peptides can reduce the toxic effects of intracellular ion influx associated with excitotoxicity and ion channel over-stimulation.

### Mitochondrial Targeting and Maintenance of Mitochondrial Integrity

CARPs have the capacity to target mitochondria and exert positive effects on the organelle, with potential neuroprotective outcomes. This topic has been the subject of several reviews by the developers of the mitochondrial targeting SS cationic arginine-containing tetrapeptides (240), and more recently by our own laboratory (241), and therefore will be discussed only briefly here.

After entering cells, CARPs target and enter mitochondria due to the presence in the outer and inner mitochondrial membranes of negatively charged phospholipids (e.g., cardiolipin, PIP2), and because of the mitochondrial transmembrane potential (ΔΨm). It is also possible that electrostatic interactions of CARPs with negatively charged free mitochondrial DNA contributes to the retention of the peptides in the organelle. At the site of the outer mitochondrial membrane, CARPs can inhibit the toxic influx of calcium into mitochondria, possibly by perturbing ion channel receptors (e.g., MCU, VDAC, NCX) and other membrane proteins (e.g., mitochondrial permeability transition pore proteins) responsible for the movement of calcium ions into mitochondria.

CARPs can also perturb other outer membrane proteins that are detrimental to mitochondrial function and cell survival. For example, CARPs interfere with BAX, the mitochondrial permeability transition pore and other pro-apoptotic proteins that localize to the outer mitochondrial membrane during cell death or interfere with proteins that promote mitochondrial fission and mitophagy. Inhibition of mitochondrial fission enables maintenance of mitochondria as filamentous structures, which enables toxic products generated by dysfunctional mitochondria to be distributed over a large organelle volume and thereby minimizing any detrimental effects. Furthermore, due to their interactions with cardiolipin, CARPs assist in stabilizing and preserving cristae architecture and the electron transport chain with positive effects on ATP maintenance, reduced reactive nitrogen species/reactive oxygen species (ROS/RNS) generation, as well as maintenance of cytochrome c native tertiary structure, function, oxidation state and location within the inner mitochondrial membrane. In addition, the anti-oxidant and free radical scavenging properties of CARPs (discussed below) would also have positive influences in reducing the toxic effects of excessive ROS/RNS generation by mitochondria during cellular stress.

### Anti-oxidant and Free Radical Scavenging Properties

Due to the amino acid arginine, CARPs are likely to act as anti-oxidant and/or free radical scavenging molecules in their own right. Although L-arginine is utilized by nitric oxide synthase as a substrate for nitric oxide generation, which is a key regulator of endothelial cell function and blood flow, the amino acid has other properties. Arginine is unique in possessing a N-terminal guanidinium head group (Figure 1A) and guanidinium containing small molecules are known to possess properties that mitigate the effects of oxidative stress. For example, L- and D-arginine, along with aminoguanidine, methylguanidine, guanidine, and creatine, which are all structurally related to arginine have the ability to scavenge one or more of the following reactive molecules: superoxide, peroxynitrate, hydroxyl radicals, hydrogen peroxide, hypochlorous acid, and breakdown products of lipid peroxidation (e.g., reactive aldehydes: malondialdehyde and 4-hydroxynonenal) (242–249).

The anti-oxidant properties of aminoguanidine, which also possesses neuroprotective actions (Supplementary Table 3), were demonstrated *in vitro* with the agent reducing rat retinal Muller cell hydrogen peroxide oxidant induced apoptosis, ROS production and lipid peroxidation, and *in vivo* by reducing the level of lipid peroxides in the vitreous of diabetic rats (246). Furthermore, both L- and D-arginine reduced oxidative impairment to myocardial contractility of perfused rat hearts subjected to oxygen radical generation (249). L- and D-arginine and L- and D-arginine polymers (e.g., poly-arginine R9) have beneficial effects on vascular endothelial cell and cardiovascular function and have anti-atherosclerotic properties (242, 250–252). These positive effects have also been observed with D-arginine containing peptides and therefore are likely to be independent of the nitric oxide pathway, as D-arginine is not readily metabolized by nitric oxide synthase. In support of this, in *Caenorhabditis elegans*, which lacks nitric oxide synthase (NOS), exposure to exogenous L-arginine prolongs worm lifespan under oxidative stress growth conditions (253).

Szeto-Schiller (SS) peptides are short tetrapeptides with alternating basic (e.g., arginine, lysine, or ornithine) and aromatic (e.g., tyrosine, dimethyltyrosine, tryptophan, or phenylalanine) amino acids, but usually containing at least one arginine and one tyrosine or dimethyltyrosine residue (240). Several SS peptides (e.g., SS-31, SS-20, mCPP-1; Table 1) have demonstrated anti-oxidant properties by way of reducing ROS levels in cells grown under normal or oxidative stress conditions. Whereas, the anti-oxidant action of SS peptides has been attributable to the tyrosine and dimethyltyrosine residues, based on the free radial scavenging properties of guanidinium containing molecules it is likely that the arginine residue also contributes to the anti-oxidant property of SS peptides.

Larger CARP's also have anti-oxidant and lipid peroxidation reducing properties. A lactoferrin derived peptide f8 (GRRRRSVQWCAVSQPEATKCFQWQRNMRKVRGPPVSCIKRDSPIQCIQ; net charge +8.7) and a casein derived peptide f12 YPYYGTNLYQRRPAIAINNPYVPRTYYANPAVVRPHAQIPQRQYLPNSHPPTVVRRP; net charge +7.2) have demonstrated anti-oxidant activity in an *in vitro* free radical scavenging assay (254). While both f8 and f12 are large peptides with interspersed arginine residues, molecular modeling revealed the peptides display a configuration with a highly cationic electrostatic surface, with arginine residues facing on the outside of the peptide. In addition, the human cathelicidin anti-microbial peptide LL-27 (LGDFFRKSKEKIGKEFKRIVQRIKDFL; net charge +5) inhibits the oxidation of low density (LDL) and high density (HDL) lipoproteins and can reduce fatty acid hydroperoxides in *in vitro* oxidation models (255). As demonstrated with protamine, CARPs also bind negatively charged oxidized-LDLs, inhibit their engagement to the lectin-like oxidized low-density lipoprotein receptor-1 (LOX-1), which can stimulate intracellular signaling cascades detrimental in ischemia-reperfusion cerebral injury (256, 257). Similarly, the cathelicidin PR-39 (RRRPRPPYLPRPRPPPFFPPRLPPRIPPGFPPRFPPRFP; net charge +10) protects HeLa cells from apoptotic cell death induced by the oxidizing agent tert-butyl hydroperoxide (198), and inhibits hypoxia induced cell death of endothelial cells (199).

Another mechanism how CARPs can reduce oxidative stress is by inhibiting the activity of the plasma membrane superoxide generating enzyme complex nicotinamide adenine dinucleotide phosphate oxidase (NADPH oxidase), with one study indicating inhibition is associated with the presence of poly basic amino acid consisting of arginine, lysine or histidine motifs (258). The CARPs PR-39, PR-26 (amino acids 1–26 of PR-39; net charge +8), gp91ds-tat (Table 2), and TAT-NR2B9c all inhibit NADPH oxidase function or superoxide generation in cell free systems and/or in different cells both *in vitro* and *in vivo* (259–261). The NADPH oxidase complex consists of 5 subunits with PR-39 and PR-26 binding to the SH3 (SRC homology 3) domain within the p47^phox^ subunit, which disrupts binding to the p22^phox^ subunit (261). The gp91ds-tat is derived from the NADPH oxidase Nox2 cytosolic B loop (mouse Nox2; amino acids 86–94) subunit and was designed to inhibit Nox2 interacting with p47^phox^ (260). TAT-NR2B9c inhibits the generation of superoxide and phosphorylation of p47^phox^ in cultured neurons exposed to NMDA (259). It was concluded that inhibition of NMDA receptor-PDS-95 mediated signaling by TAT-NR2B9c prevented phosphorylation of p47^phox^ and activation of NADPH oxidase. However, for both the gp91ds-tat and TAT-NR2B9c peptides a direct inhibitory action on NADPH oxidase associated with the arginine content and positive charge of the peptides cannot be ruled out. In addition, given all four peptides have cell-penetrating properties, it is possible that these and other CARPs disrupt the membrane assembly of NADPH oxidase units within the plasma membrane. Since superoxide generation is also associated with inflammatory responses, inhibition of NADPH oxidase activity would also contribute to the anti-inflammatory properties of CARPs.

While the anti-oxidant properties of CARPs need to be further investigated, available evidence suggests that arginine residues within the peptide have the potential to exert anti-oxidant and/or free radical scavenging actions. Furthermore, it could be hypothesized that the multiple arginine resides within CARPs will act as a multivalent anti-oxidant compound, which depending on the number and arrangement of the guanidinium moieties would provide considerably more potency per molecule than arginine alone or a molecule containing a single guanidine moiety.

### Methylglyoxal Scavenging and Glycation End-Products

Glyoxal compounds are highly reactive cell permeable dicarbonyls produced predominantly as a by-product of glycolysis, and are precursors in the formation of advanced glycation end-products (AGEs). An important dicarbonyl with respect to cellular toxicity is methylglyoxal, which reacts irreversibly with arginine and lysine residues and reversibly with cysteine resides on proteins causing functional impairment (262). Methylgyloxal also reacts with nucleic acids and lipids, and its production is associated with oxidative stress and ROS generation. The glycation of arginine and lysine by methylglyoxal forms the AGEs hydro-imidazolone, methylglyoxal-hydroimdazolone 1 (MG-H1), argpyrimidine, and MG-derived lysine dimer. In addition, to altering protein function, AGE-modified proteins interact with AGE receptors (RAGE), which stimulate the expression of inflammatory genes and ROS generation. AGEs are increased in diabetes, vascular disease, cerebral ischemia, renal failure, aging and chronic disorders, such as Alzheimer's disease, Parkinson's disease and liver cirrhosis. Methylgyloxal can affect mitochondrial function, up-regulate vascular adhesion molecules (e.g., P-selectin and E-selectin) that contribute to leucocyte adhesion (263), and cause glycation of the BBB vascular tight junction protein occluding (264) and the basement membrane extracellular protein fibronectin (265) resulting in altered endothelial function.

Given that methylglyoxal readily targets basic amino acids, it is likely that arginine (and lysine/cysteine) residues within CARPs react with and act as methylglyoxal scavengers, thereby reducing their toxic effects on intra- and extra-cellular proteins, and RAGE activation. Methylgloxal is normally detoxified by the glyoxalase system (glyoxalase-1 and glyoxalase-2), which utilizes glutathione as a co-factor, however neurons are particularly susceptible to methylgloxal due to the high glycolytic activity of the brain and the reduced capacity of the glyoxalase system in neurons. The capacity of the glyoxalase system in the brain deceases with age, especially after the fifth decade of life, and an increase in MG-H1 modified mitochondrial proteins is linked to aging and increased ROS production (266); increasing glyoxalase capacity in *C. elegan* increases life span in this organism (267). Furthermore, any additional stress within the brain as occurs in cerebral ischemia/reperfusion, as well as in chronic neurological disorders is likely to lead to an excessive production of methylgloxal and/or reduced capacity to detoxify methylgloxal.

Importantly, arginine and other guanidinium containing molecules (e.g., aminoguanidine, metformin) have the capacity to scavenge methylglyoxal and prevent AGEs (268, 269). For example, at one stage aminoguanidine was being developed as a therapeutic agent for the prevention of AGEs in diabetes, and both L- and D-arginine can effectively scavenge and attenuate the harmful effects of methylgloxal on cultured endothelial cells (270). However, it is considered that small molecule methylglyoxal scavengers are not sufficiently potent and/or suffer from short half-lives to be effective *in vivo*. In contrast, arginine-containing penta-peptides peptides (CycK[Myr]RRRRE; Cyc, cyclic peptide; Myr, myristic acid; and myr-KRRRRE; net charge +4) also possess methylglyoxal scavenging activity (271), and CycK(Myr)RRRRE prevents methylglyoxal induced pain in mice, and is being considered as a therapy for pain and other diabetic complications associated with methylglyoxal toxicity (271).

### Inhibition of Matrix Metalloproteinase Activation and the Proteasome

Another mechanism whereby CARPs may exert a neuroprotective effect is by their ability to indirectly prevent the activation of matrix metalloproteinases (MMPs) by inhibiting proprotein convertase (PC) activation. Proprotein convertase consists of a family of proteolytic enzymes that cleave inactive proteins, including MMPs into an active state. Poly-arginine peptides and other CARPs are potent inhibitors of convertases, such as furin, PC1, PC4, PC5/6, and PC7 (272–276).

Furin is a ubiquitously expressed convertase that is regulated by hypoxia-inducible factor-1 (HIF-1) (277) and up-regulated in the ischemic brain (278, 279), and can activate MMP2, MMP3, and MMP14 (280). Furthermore, MMP3 can activate MMP1, MMP7 and MMP9, and MMP14 can activate MMP2 and MMP2 can activate MMP9 (281, 282). Significantly, following ischemic stroke MMP2, MMP3, MMP7, MMP9, and MMP14 are either up-regulated or activated in the brain. Moreover, MMP activation is associated with degradation of the neurovascular unit and BBB disruption, which in turn can result in cerebral edema, leukocyte infiltration and secondary hemorrhage after ischemia (279, 281, 282). It is also possible that proprotein convertases have other protein substrates, which when activated are potentially neuro-damaging, however this is an area that has as yet not been explored.

The ability of poly-arginine peptides to inhibit proprotein convertases, similar to peptide neuroprotection (15), increases with increasing polymer length (e.g., R9 > R8 > R7 > R6) (272). In addition, convertase inhibition is not stereospecific with both to L- and D-isoform peptides having the capacity to inhibit enzyme activity. The electrostatic interaction between CARPs and the negatively charged surface of convertases is believed to be the mechanism responsible for the inhibitory actions of the peptides. Interestingly, a penetratin-fused peptide (P-IQACRH: RQIKIWFQNRRMKWKK-IQACRG; net charge +7.9) that mimics the active site of caspases 1, 2, 3, 6, 7 and 14 and acts as a competitive inhibitor for these enzymes, inhibited caspase and MMP9 activation following NMDA-induced excitotoxicity in an *in vivo* retinal ganglion cell injury model (283). However, the peptide also reduced NMDA-induced retinal ganglion cell death in culture and *in vivo*, and hence it is possible that the anti-excitotoxic properties of P-IQACRH, rather than a direct down-stream inhibition of caspases and MMP9 was responsible for blocking the activation of the enzymes. The P-IQACRH study highlights the caution that is needed when analyzing the neuroprotective actions of CARPs.

CARPs can also inhibit other proteolytic enzymes, such as cathepsin C (284) as well as, the activity of the proteasome (285–289). Importantly, treatments known to inhibit the proteasome, which is responsible for the degradation of short-lived cytosolic proteins, is known to reduce the severity of brain injury after stroke (290–293).

With respect to proteasomal inhibition, the CARP PR-39 (see above) can reversibly bind to the α7 subunit of the 26S proteasome and block degradation of the nuclear factor-κB (NF-κB) inhibitor protein IκBα. Interestingly, studies utilizing PR-39 indicate that proteasomal inhibition occurs via a unique allosteric, reversible and substrate selective mechanism without inhibiting overall-proteasome proteolytic activity, which in itself could be deleterious by interfering with normal cellular processes. In contrast, mild levels of proteasome inhibition can induce a protective pre-conditioning response that can protect cells from oxidative stress (294). Similarly, ischemic pre-conditioning, which can reduce brain injury following stroke is associated with proteasomal inhibition (293). PR-39 abolished NF-κB-dependent gene expression in cultured endothelial cells exposed to TNF-α, and in the pancreases and hearts of mice following induction of acute pancreatitis and myocardial infarction, including the up-regulation of vascular cell adhesion molecule-1 (VCAM-1) and intercellular adhesion molecule-1 (ICAM-1) (285). Other studies have also demonstrated that PR-39 can reduce infarct size and have beneficial effects on microvascular cells in myocardial reperfusion injury models by blocking proteasome-mediated degradation of IκBα (200).

In contrast to blocking activation of the NF-κB, inhibition of the proteasome is likely to enhance activation of the transcription factor HIF-1, which is considered one of the most critical adaptive gene expression responses to low oxygen concentrations. Hypoxia-inducible factor-1 consists of the HIF-α and HIF-β subunits, the former undergoing proteasomal degradation during normoxia, and the latter being constitutively expressed. Therefore, inhibition of the proteasome will enhance and/or prolong HIF-1 activation in the brain during and following cerebral ischemia, thereby enhancing any neuroprotective actions of the transcription factor. For example, PR-39 was demonstrated to inhibit the proteasome-dependent degradation of HIF-α and stimulate angiogenesis by accelerating the formation of vascular structures in cultured endothelial cells and *in vivo* in the myocardium (295). A similar effect was demonstrated in the brain after stroke with a small molecule proteasome inhibitor resulting in accumulation of HIF-α and enhanced angio-neurogenesis (291).

Inhibition of the proteasome can also enhance the activity of the transcription factor nuclear factor E2-related factor 2 (Nrf2), which regulates the expression of multiple cytoprotective genes, particularly those involved in mitigating oxidative stress (e.g., HO-1, SOD1, NAD[P]H dehydrogenase, glutathione S-transferase) (296). Under normal conditions, cytoplasmic Nrf2 is bound to kelch-like ECH-associated protein 1 (Keap1), in which it is subject to proteasomal degradation, however oxidative stress disables keap1, allowing Nrf2 to accumulate, translocate to the nucleus and activate gene expression. Interestingly, a Nrf2 amino acid derived sequence (LQLDEETGEFLPIQ) has been developed that disrupts the Nrf2-Keap1 interaction, and when fused to TAT (TAT-14: YGRKKRRQRRR-LQLDEETGEFLPIQ; charge +4) or R7 (7R-ETGE: RRRRRRRR-LQLDEETGEFLPIQ; net charge +4) has been demonstrated to activate Nrf2 and cytoprotective gene expression in THP-1 monocyte and RAW 264.7 macrophage cell lines (297, 298). Furthermore, the TAT-14 peptide modified to contain a calpain cleavage sequence (TAT-CAL-DEETGE: Table 2) increased Nrf2-regulated gene expression in the brain (299) and is beneficial when administered to rodents after global cerebral ischemia and TBI (154, 299). It remains to be determined if the Nrf2 peptides are activating Nrf2 by directly disrupting the Nrf2-Keap1 interaction or by inhibiting the proteasome. Also of interest is the demonstration that in rats, oral treatment with arginine, resulted in the up-regulation of proteins associated with the Nrf2 pathway in liver and plasma (300).

### Reducing the Inflammatory Response

Whereas, few studies have specifically examined neuroprotective CARPs in the setting of neuro-inflammation, this class of peptide has well-established anti-inflammatory properties that are potentially beneficial in neurodegenerative disorders. It is likely CARPs exert differential effects on the immune response by targeting both the CNS and peripheral immune responses by several mechanisms. As explained above, the ability of CARPs to inhibit the proteasome will reduce NF-κB activation and the expression of genes involved in pro-inflammatory pathways. Interestingly, the CARP AIP6 (RLRWR; net charge +3) can inhibit NF-κB activity by an alternative mechanism, by binding to and blocking NF-κβ p65 sub-unit binding to DNA and inhibiting its transcriptional activity (301). The p65 subunit is a negatively charged protein, and hence it is possible that CARPs have the capacity to interfere with this NF-κβ sub-unit through an electrostatic interaction. Similarly, because CARPs can interfere with cell surface receptors levels and/or function, it is also possible they reduce the inflammatory response associated with ligands (e.g., cytokines, chemokines, intracellular molecules) binding to receptors on immune cells.

Proteins regulated by NF-κB and involved in the inflammatory response include cytokines (e.g., IL-1, TNF-α), chemokines (e.g., MCP-1, CXCL1) and vascular adhesion molecules (e.g., ICAM-1, VAM-1) (302). To this end, NF-κB is responsible for up-regulating cerebral vascular adhesion molecules VCAM-1 and ICAM-1, which during cerebral reperfusion promotes macrophage and neutrophil infiltration into the brain. Although the ability of CARPs to reduce vascular adhesion molecule expression in the cerebral vasculature has not been examined, PR-39 can reduce VCAM-1 and ICAM-1 protein levels in heart tissue following myocardial infarction in mice and in cultured vascular endothelial cells following exposure to TNF-α (285), and leukocyte adhesion to rat mesenteric venules after ischemia and reperfusion (303). In addition, the TAT peptide reduces the production of multiple cytokines (e.g., G-CSF, IL-6, MIP1α, TNF-α, and IFN-γ) in cultured human lung epithelial cells following protein kinase C stimulation by phorbol 12, 13-dibutyrate, a stimulus associated with NF-κβ activation (304). In line with the ability of CARPs to inhibit the proteasome, TAT reduced degradation of the NF-κβ inhibitory subunit IK-κβ in lung epithelial cells following protein kinase C activation. Similarly, AIP6 demonstrated anti-inflammatory effects in cultured activated macrophages by decreasing TNF-α and prostaglandin-E secretion and in a mouse model of paw inflammation reduced levels of TNF-α, IL-1β, and IL-6 protein in affected tissue (301).

CARPs can also bind to oxidized phospholipids (e.g., ox-LDLs) which are known pro-inflammatory molecules and play an important role in atherosclerosis and other inflammatory disorders. Due to the high lipid content of the brain, any conditions that increase oxidative stress will generate oxidized phospholipids. Binding of CARPs to oxidized phospholipids is believed to enhance their clearance, as well as reduce their inflammatory potential and inhibitory effects on anti-oxidant enzymes associated with lipoproteins and the cell membrane (255, 305). The CARP E5 (Ac-SHLRKLRKRLLRDADDKRLA-NH_2_; net charge +6) was demonstrated to bind oxidized phospholipids and inhibit their pro-inflammatory function in human blood (305). In addition, pre-treatment of macrophage (RAW264.7) and endothelial (HUVEC) cell lines with the LL-27 reduces pro-inflammatory gene expression, whereas pre-incubation of oxidized phospholipid with the peptide prior to administration to mice reduces serum IL-6 and TNF-α levels (255). Similarly, the Apolipoprotein E (ApoE) protein derived CARP Ac-hE18A-NH_2_ (Ac-RKLRKRLLRDWLKAFYDKVAEKLKEAF-NH_2_; net charge +6) can bind bacterial lipopolysaccharides (LPS) and reduce its inflammatory (e.g., TNF-α, IL-6 production) inducing properties in human blood and primary leukocytes and a monocyte cell line (306). Ac-hE18A-NH_2_ can also inhibit LPS-induced VCAM-1 expression, and reduce monocyte adhesion in HUVECs, as well as the secretion of IL-6 and monocyte chemoattractant protein-1 (MCP-1) from THP-1 monocyte cells exposed to LPS (307). Also, the CARP TAT-14 (see above), which while developed to activate Nrf-2, reduces TNF-α production in THP-1 monocyte cells following LPS stimulation (297).

Other ApoE derived peptides have also demonstrated anti-inflammatory properties. The two almost identical ApoE derived peptides, ApoE-133–150 (ApoE-133–150: Ac-LRVRLASHLRKLRKRLLR-NH_2_; net charge +8.1) and COG-133 (Table 1) suppress cytokine expression (IL-8 or TNF-α) and other inflammatory mediators (e.g., COX or NO) in THP-1 monocytes or BV-2 microglia cells stimulated with LPS (308, 309). COG-133 treatment can suppress systemic and brain levels of TNF-α and IL-6 in mice after LPS administration (36). The COG112 peptide, which comprises COG133 fused to the CCPP penetratin (Table 2) inhibits the inflammatory response in mouse models of pathogen or injury induced colitis by reducing several pro-inflammatory mediators. For example, COG112 attenuated cytokine and chemokine expression, iNOS expression and nitric oxide production in mouse colon epithelial cell cultures and in colon tissue in mice following exposure to *Citrobacter rodentium* (310, 311). It was also demonstrated that COG112 inhibited NF-κB activation in colon cells and tissue following bacterial stimulation (310, 311).

The CARP PACAP38 (Table 3), which is derived from the neuropeptide pituitary adenylate cyclase-activating polypeptide (PACAP) binds the adenylate-cyclase-activating receptor stimulating adenylate cyclase and subsequently increases intracellular cAMP, which is a signaling molecule important in many biological processes. PACAP38 is a predominant cleavage product of PACAP, which exerts several functions within the CNS, including acting as a neurotransmitter and neuromodulator and modulating inflammatory responses. The peptide has cell-penetrating properties (312), is widely distributed within the CNS and has neuroprotective actions in excitotoxicity, retinal ischemia, stroke and traumatic brain injury models (313–318). With respect to its anti-inflammatory actions, PACAP38 can reduce the activation of cultured primary microglia to hypoxia by inhibiting induction of nitric oxide, iNOS, and p38 as well as reducing TNF-α secretion (315). Furthermore, following traumatic brain injury, PACAP38 treatment reduces cerebral inflammation by reducing toll-like receptor-4 (TLR-4) up-regulation, and its downstream mediators. For example, treatment reduced TNF-α and IL-1β levels, reduced NF-κβ p65 sub-unit levels in nuclei, and increased levels of the NF-κβ inhibitory subunit IκB-α in the brain (318). Additionally, PACAP38 ablated TLR-4 up-regulation in the brain and in BV-2 microglia following exposure to the TLR-4 agonist LPS (319). Similarly, the LL-37 anti-microbial CARP (LLGDFFRKSKEKIGKEFKRIVQRIKDFLRNLVPRTES; net charge +6) attenuates activation of cultured dendritic cells to different TLR ligands (320).

The CARP dRK (rrkrrr; net charge +6; lower case indicates D-isoform amino acids) was identified based on its ability to block the interaction between VEGF and the VEGF receptor. The dRK peptide can reduce TNF-α and IL-6 production in normal peripheral blood monocytes and synovial fluid mononuclear cells of rheumatoid arthritis patients following VEGF stimulation (321). In a mouse model of collagen-induced arthritis, dKR reduced paw inflammation and serum levels of IL-6 (321). While it was believed that dKR was directly inhibiting the pro-inflammatory effects of VEGF and its receptor, it is possible the peptide was inhibiting VEGF induced activation of NF-κβ. In a different collagen-induced arthritis mouse model, the CARPs IG-19 (IGKEFKRIVQRIKDFLRNL-NH_2_; net charge +5) and IDR-1018 (Table 1) both reduced the number of limbs affected, and IG-19 reduced overall disease severity (322). Subsequent examination of IG-19 treated mice revealed reduced serum levels of the pro-inflammatory cytokines TNF-α and IFN-γ and reduced cellular infiltration and cartilage degradation in arthritic joints. Interestingly, in a more recent study, IDR-108 prolonged anti-inflammatory TGFβ gene expression and suppressed early pro-inflammatory IL-1β gene expression levels in a human endothelial cell line (EA.hy926) cultivated in a high glucose environment to induce cell stress (323). In another study, treatment of arthritic mice with a peptide developed to mimic the action of Bcl-2 homology 3 (BH3) domain-only proteins (TAT-BH3: Ac-RKKRR-O-RRR-EIWIAQELRRIGDEFNAYYAR; net charge +6) ameliorated arthritis development and reduced the number of myeloid cells in the affected joint (324).

The CARP R9-SOCS1-KIR (RRRRRRRRR-DTHFRTFRSHSDYRRI; net charge +11.2) was developed to inhibit suppressor of cytokine signaling 1 (SOCS1) signaling, which can result in JAK/STAT or NF-κβ activation. This peptide blocked the activation and nuclear translocation of STAT1α, STAT3, and NF-κB p65 and inflammatory effects induced by IFN-γ, TNF-α, and IL-17A in the ARPE-19 human retinal pigment epithelial cell line (325). Topical delivery of R9-SOCS1-KIR also reduced inflammatory cell infiltration into the eye in a mouse model of experimental autoimmune uveitis. Furthermore, in a mouse model of *Pseudomonas aeruginosa* induced keratitis, R9D (Table 1) treatment reduced disease severity and concentrations of corneal TNF-α, IFN-γ, IL-10, and GM-CSF (326).

The cationic arginine-rich human beta-defensin derived peptide hBD3-3 (GKCSTRGRKCCRRKK; net charge +8) has demonstrated *in vitro* and *in vivo* anti-inflammatory actions. In a macrophage cell line (RAW264.7) pre-treatment with hBD3-3 reduced iNOS, TNF-α, and IL-6 protein expression (327). In addition, mice treated with hBD3-3 and injected with LPS had reduced plasma levels of TNF-α and IL-1β and reduced neutrophil infiltration into lung regions affected by LPS induced inflammation. Finally, as NF-κβ activation is involved in iNOS, TNF-α and IL-6 expression, studies in RAW264.7 cells revealed that hBD3-3 significantly inhibited degradation of the NF-κβ inhibitory subunit IκB-α, as well as the translocation of the NF-κβ p65 subunit to the nucleus.

CARPs may also inhibit inflammation by reducing activation of components of the complement system. Protamine (Table 1) and large poly-L-arginine peptides antagonize complement protein C5a binding to its receptor C5aR1 (or CD88) in leukocytes (328). C5aR is a transmembrane G-protein-coupled receptor expressed on neutrophils, monocytes, eosinophils, and non-myeloid cells, including liver cells and alveolar and kidney tubular epithelial cells, some classes of neurons and microglia and astrocytes. Importantly activation of the complement system following stroke/cerebral ischemia and other neurological conditions is associated with unfavorable outcomes and inhibition of C5a improves outcomes (329). Antagonism of the C5aR is thought to be due to an electrostatic interaction between the CARP and anionic sites within the receptor (328).

In some situations, CARPs may induce pro-inflammatory responses. For example, a large poly-arginine peptide (R100; 100-mer, 12.5–13.5 kDa) can bind to TLR-4 and induce cytokine and interferon gene expression in mouse splenocytes comprising mostly of B-cells, but also T-cells and monocytes (330). The peptides ApoE-133–150 and IDR-1018 increase secretion of the cytokine MCP-1 in human blood mononuclear cells (309). In one study, IDR-1018 also increased neutrophil adherence to EA.hy926 endothelial cells and promoted neutrophil migration and cytokine production (e.g., IL-8, MCP-1, MCP-3) (331).

### Pro-survival Signaling

Due to the ability of CARPs to interact with cell surface receptors it appears they also have the capacity to stimulate receptor mediated pro-survival signaling pathways. The best example of CARP pro-survival signaling has been demonstrated with apelin peptides.

Apelin is a highly conserved arginine-rich peptide first identified in 1998 following its isolation in bovine stomach extracts. The peptide is expressed as a 77 amino acid preprotein, which can be processed into at least three bioactive carboxy-terminal fragments including apelin-36, apelin-17, and apelin-13 (Table 3). All apelin peptides can bind the G-protein coupled apelin receptor (originally named APJ) and induce cell signaling (332), with positively charged arginine and lysine resides in apelin, and negatively charged aspartate and glutamate resides in the extracellular N-terminal region of the apelin receptor important for receptor binding and internalization (333, 334). Apelin peptides and the apelin receptor are widely expressed throughout the body including brain, heart, adipose, skeletal muscle, kidney, and lung. The apelin/apelin receptor system regulates cardiac and vascular function, glucose metabolism, fluid homeostasis, cell survival, and angiogenesis. Other CARPs including poly-arginine peptide R9D and protamine can bind to the apelin receptor (333, 335). Interestingly, pre-treatment of cells with R9D and protamine appears to inhibit subsequent apelin receptor signaling. However, this is likely due to the R9D and protamine peptides desensitizing the receptor or inducing receptor internalization because pre-treatment of cells with apelin peptides also decreases apelin receptor cell signaling (336). The apelin receptor can dimerise with the κ-opioid G-protein-coupled receptor (KOR) and bind both apelin and dynorphin A peptides (Table 3) and activate extracellular signal–regulated kinase 1/2 (ERK1/2) signaling (337).

Established signaling events activated by the apelin receptor are the AMP-activated protein kinase (AMPK), ERK1/2 and phosphatidyl inositol 3-kinase/protein kinase B (PI3K/AKT) pathways (338–340). The AMPK pathway is a major energy sensing system that monitors for low levels of energy molecules, such as ATP and AMP to induce cellular adaptive metabolic changes to preserve and better utilize remaining energy substrates and maintain mitochondrial function. In addition, AMPK can activate the transcription factor Nrf2, resulting in the expression of anti-oxidant proteins. The ERK pathway has diverse actions including cell survival mediated by the expression of pro-survival proteins (e.g., BCL2) and inhibition of pro-apoptotic proteins (e.g., BAD). Similarly, the PI3K/AKT pathway promotes cell survival by targeting and phosphorylating proteins that regulate cell death and survival, cell migration and metabolism and angiogenesis.

With respect to neuroprotection, apelin peptides reduce intracellular calcium influx and neuronal death following NMDA receptor mediated excitotoxicity (163–166, 341), and improve outcomes in animal stroke, perinatal hypoxia-ischemia, traumatic brain injury, intracerebral hemorrhage and Alzheimer's disease models (see Table 3). The neuroprotective mechanism of action of apelin peptides have been attributed to AMPK, ERK, and/or AKT mediated signaling by inhibiting apoptosis, suppressing inflammation, reducing ER stress, preserving BBB integrity and stimulating angiogenesis (163, 166–170, 340–342), as well as mechanisms independent of apelin receptor signaling (164, 165).

The endogenous CARP toddler (also known as elabela/apela; QRPVNLTMRRKLRKHNCLQRRCMPLHSRVPFP; net charge +9.1) can also bind the apelin receptor and induce ERK signaling (343). Whereas, an anti-microbial CARP SR-0379 (MLKLIFLHRLKRMRKRLKRK; net charge +11) can stimulate ERK and AKT phosphorylation in dermal fibroblasts via a cell surface integrin receptor (344). In addition, the anti-microbial LL-37 peptide can bind to cell surface receptors in different cells, and activate downstream ERK, AKT, or P38 signaling (345, 346). Finally, protamine and polycationic arginine and lysine peptides interact with and enhance the EGF receptor tyrosine kinase activity and thereby enhance cell signaling activated by the receptor (347, 348).

### Inhibiting Protein Aggregation in Neurodegenerative Disorders

Protein misfolding can lead to protein aggregation, and the accumulation of specific protein oligomers, aggregates and fibrils is the hallmark of several chronic neurodegenerative disorders, such as Alzheimer's disease (e.g., Aβ peptide, tau), Parkinson's disease (e.g., α-synuclein), Huntington's disease (e.g., Huntingtin), and amyotrophic lateral sclerosis (e.g., SOD1). Arginine is a common additive to protein solutions to facilitate protein folding, and to help maintain protein stability and inhibit protein aggregation (349, 350). Since arginine can stabilize proteins and inhibit self-aggregation, it is possible CARPs can also reduce protein misfolding and aggregation, and is a mechanism through which CARPs may be beneficial in animal models of human neurodegenerative disorders associated with proteinopathies.

The guanidinium head group of arginine and the ability of arginine to self-associate to form clusters (*n*-mers; *n* > 2) is considered a critical mechanism responsible for suppressing protein aggregation (349). Arginine clusters associate with the surface of proteins, namely aromatic (e.g., tryptophan) and negatively charged (e.g., glutamate) amino acid residues via cation-π interactions and hydrogen bonding, respectively. The interaction of arginine clusters with hydrophobic residues not normally exposed in the native state stabilizes partially unfolded proteins and act to “crowd around” proteins to prevent aggregation (350). Hence, it is conceivable that CARPs, such as poly-arginine molecules due to their multivalent arginine arrangement behave in a similar fashion to arginine clusters to prevent protein aggregation. For example, CARPs that inhibit Aβ oligomer formation, which is considered neurotoxic include KLVFFRRRRRR (net charge +7) and R5 (RRRRRR: net charge +5) (351), 15M (Ac-VITNPNRRNRTPQMLKR-NH_2_: net charge +5) (352) SRPGLRR (net charge +3) (353), RR-7-animo-4-trifluromethylcoumarin (net charge +3) (354) RI-OR2-TAT (Ac-rGffvlkGrrrrkkrGy-NH_2_: charge +9) (355) R8-Aβ (25–35) (rrrrrrrr-gsnkgaiiglm: net charge +10) (356), and the related D3 and RD2 peptides (Table 1) (28, 29). In a mouse model of Alzheimer's disease, R9 administered subcutaneously over 4 weeks, decreased brain Aβ deposits by 15%, albeit the reduction was not statistically significant (357). In addition, poly-arginine (5–15 kDa; 32–96-mers) can inhibit the aggregation of a tau mutant protein (P301L) commonly associated with tauopathy (358), and the CARPs P42-TAT (Table 2) (118) and TAT-P110 (Table 2) (149) inhibit aggregation of mutant Huntingtin protein. Interestingly, several of the key proteins that accumulate in the CNS in chronic neurodegenerative disorders are negatively-charged (e.g., Aβ 1–42: −2.7; tau: −6.2; α-synuclein: −8.9; Huntington: −59.7; SOD1: −5.5), which would increase their electrostatic affinity to positively-charged molecules, and make them ideal therapeutic targets for CARPs.

## Endogenous CARPs and Neuroprotection

The endogenous PACAP38 peptide is a member of the secretin/glucagon/growth hormone-releasing hormone superfamily, and its neuroprotective properties have been discussed above. Dynorphins are widely distributed in the CNS and consist of two main peptides dynorphin A (Table 3) and dynorphin B (Table 3) that bind the κ-opioid receptor to induce analgesia (359). Interestingly, many other synthetic CARPs also have analgesic properties (see Tables 1, 2). Dynorphin A and dynorphin B are synthesized as the precursor protein predynorphin, which is then proteolytically cleaved to the smaller peptides. The dynorphin A peptide has also been shown to be neuroprotective in a rat stroke model (177).

Similarly, different classes of endogenous anti-microbial peptides (e.g., defensins, cathelicidins, bactenecin) have been derived from mammals, many of which are cationic and arginine-rich. Anti-microbial peptides are mainly produced by leukocytes and act as a defense against bacteria, fungi and viruses, and act either directly or by modulating inflammatory responses. Interestingly, several cationic arginine-rich anti-microbial peptides have also been shown to have neuroprotective properties in stroke, perinatal hypoxia-ischemia and traumatic brain injury animal models (Table 2).

## Compounds Containing the Guanidinium Moiety and Neuroprotection

As mentioned above, arginine is unique in possessing a guanidinium head group, and most likely the critical element imparting the neuroprotective properties of CARPs. It is therefore not surprising that compounds containing the guanidinium moiety including arginine, arginine-based NOS inhibitors (e.g., L-NNA, L-NAME), the drugs metformin, phenformin, amiloride, and aminoguanidine, the toxin tetrodotoxin and the endogenous neuroactive molecule agmatine have neuroprotective properties in *in vitro* neuronal injury models (e.g., excitotoxicity, oxygen-glucose deprivation) and in animal models of stroke, perinatal hypoxia-ischemia, spinal cord injury, traumatic brain injury Parkinson's disease and Alzheimer's disease (Supplementary Table 3). It is thus conceivable that CARPs and other guanidinium moiety containing small molecules, at least in part, share the same neuroprotective mechanism of actions including anti-excitotoxic properties. In support of their anti-excitotoxic properties different guanidinium moiety containing molecules have been demonstrated to inhibit voltage gated and ligand-gated ion channels (230–237). However, because CARPs are multivalent guanidinium-agents they are likely to possess greater potency at the molar level than molecules that contain only one or several guanidine moieties, and have a greater capacity to traverse cell membranes.

Metformin and phenformin are biguanindine anti-hyperglycemic agents, which have been used for the treatment of diabetes for over 50 years. Like CARPs, metformin can activate AMPK signaling, target and suppress mitochondrial ROS production, limit calcium induced intracellular toxicity, scavenge methylglyoxal and reduce neuroinflammation (269). Aminoguanidine can also scavenge methylglyoxal and other dicarbynols (25). Agmatine is an endogenous divalent cationic guanidine. In the brain it is considered a putative neurotransmitter, in which it can be released from synaptic vesicles following membrane depolarization. It binds to various receptors (e.g., α2 adrenergic receptor) and can block NMDA receptors and other cation ligand-gated channels, with studies indicating that agmatine binds to the receptor near the channel pore and that the guanidinium group is critical for binding (360).

Arginine-based nitric oxide inhibitors, such as L-NNA and L-NAME are commonly used in *in vitro* excitotoxic and animal stroke studies to determine the neurodamaging role of nitric oxide over-production in neuronal death and ischemic brain tissue injury. However, given the potential anti-excitotoxic action of the guanidine moiety, it is possible that arginine-based NOS inhibitors in the setting of excitotoxicity are actually suppressing the activation of NMDA receptors and ion voltage gated channels, thereby indirectly rather than directly inhibiting NOS activation. There are several lines of evidence that support this hypothesis. Following excitotoxicity, it is difficult to imagine that by blocking neuronal nitric oxide production, but not the toxic intracellular influx of calcium is able to provide high level neuroprotection (361). In addition, arginine-based NOS inhibitors are not readily taken up by cells and possess slow NOS binding kinetics (362), but are neuroprotective when added at the same time as the excitotoxic agent, which favors an extracellular (i.e., cell surface) rather than an intracellular (i.e., NOS) mechanism of action. In addition, L-NAME is a weak NOS inhibitor, which is hydrolyzed by ubiquitous esterases to the more potent L-NNA, thus requiring additional time for the inhibitor to exert its NOS inhibitory effects.

## Concluding Remarks

There is now overwhelming evidence from experimental studies that CARPs represent a novel class of neuroprotective agent with great potential for the treatment of neurological disorders. However, only two CARPs with neuroprotective properties (TAT-NR2B9c/NA-1 and CN-105) have so far progressed to clinical trials for a neurological condition (363–365). Further studies are required to obtain a more complete understanding of the neuroprotective mechanisms of action of CARPs in acute CNS injury and chronic neurodegenerative disease models. Despite this, based on experimental studies to date it appears that CARPs have the potential to be developed as therapeutics for the treatment of a diverse range of neurological disorders including stroke, perinatal hypoxia-ischemia, traumatic brain injury and spinal cord injury as well as, epilepsy and pain, and potentially even chronic degenerative neurological disorders, such as Alzheimer's disease, Parkinson's disease, amyotrophic lateral sclerosis, and Huntington's disease. Importantly, CARPs have properties that greatly enhance the likelihood of translational success at the clinical level including possessing a pluripotent mechanism of action, the capacity to enter the CNS, and the ability to exert a broad range of beneficial extracellular, intracellular and intra-organelle effects. Based on human studies with TAT-fused peptides, such as TAT-NR2B9c/NA-1 (366), the poly-arginine peptide R9/ALX40-4C (367), and arginine-rich peptides CN-105 (365), protamine (368) and RD2 (369), it appears this class of peptide has a favorable safety profile. Moreover, our recent experimental neuroprotection study with the poly-arginine peptide R18 in a non-human primate stroke model (26), has not disclosed any neurological or other toxic effects, which also augurs well for the translational potential of other CARPs to the clinical arena.

With respect to previous studies using cationic and arginine-rich peptides including those fused to a CCPP in neuroprotective, neuroactive or cytoprotective studies we believe that due to the cofounding effects of peptide positive charge and arginine residues, the mechanisms of action of theses peptides need to be critically re-evaluated.

Finally, given that CARPs with different amino acid sequences or modifications will have different physio-chemical and biological properties, future studies should focus on examining if new CARPs with more targeted molecular mechanisms of actions can be designed to improve therapeutic efficacy for specific neurological disorders.

## Data Availability Statement

All datasets generated for this study are included in the article/Supplementary Material.

## Author Contributions

BM conceptualized and wrote the review. FM and NK provided the additional input into the content of the review and in drafting the manuscript. BM, FM, and NK contributed to the writing of the manuscript. BM prepared the figures and tables provided in the manuscript.

### Conflict of Interest

BM and NK are named inventors of several patent applications regarding the use of CARPs as neuroprotective agents. The remaining author declares that the research was conducted in the absence of any commercial or financial relationships that could be construed as a potential conflict of interest.
