# Supplementary material for: Cationic Arginine-Rich Peptides (CARPs): A Novel Class of Neuroprotective Agents With a Multimodal Mechanism of Action
Source: Front Neurol. 2020 Feb 25;11:108. doi: 10.3389/fneur.2020.00108 (PMC7052017; doi:10.3389/fneur.2020.00108)
Supplement: Supplementary file 1 [file Table_1.doc]

**SUPPLEMENTARY MATERIAL**

**Supplementary table 1** Anionic structures in cell membranes.

| **Membrane structure** | **Anionic molecule (charge)** | **Anionic moiety** |
| --- | --- | --- |
| Phospholipids | PA (-1), PS (-1), PI (-1), PIP1 (-2), PIP2 (-3), PIP3 (-4), cardiolipin (-2) | Phosphate |
| Proteoglycans; HSPG, CSPG, DSPG, KSPG | HS (-3), CS (-2), DS (-1), KS (-1) | Sulphate |
| Glycoproteins | Sialic acid (-1) | Carboxylate |
| Glycosphingolipids | Sialic acid (-1) | Carboxylate |
| Proteins/Receptors | Aspartate (-1), glutamate (-1) | Carboxylate |

HSPG - heparin sulphate proteoglycans; CSPG - chondroitin sulphate proteoglycans; DSPG - dermatan sulphate proteoglycans; KSPG - keratin sulphate proteoglycans; HS - heparin sulphate disaccharide subunit; CS - chondroitin sulphate disaccharide subunit; DS - dermatan sulphate disaccharide subunit; KS - keratin sulphate disaccharide subunit; PA - Phosphatidic acid; PS – Phosphatidylserine; PI - Phosphatidylinositol; PIP - Phosphatidylinositol phosphate; PIP2 - Phosphatidylinositol bisphosphate; PIP3 - Phosphatidylinositol trisphosphate.

**Supplementary table 2** CARPs interfering with ion channel and non-ion channel receptor function or cell surface levels.

| **Peptide name** | **Peptide sequence** | **Receptor and effect on receptor** | **Cell model** | **Reference** |
| --- | --- | --- | --- | --- |
| Protamine | PRRRRSSSRPVRRRRRPRVSRRRRRRGGRRRR | 1. PDGF receptor; blocked PDGF binding to receptor. 2. C5a receptor (CD88); Antagonised C5a binding. 3. Apelin receptor; antagonises apelin binding and signalling. 4. Glutamate receptors; reduced glutamic acid induced calcium influx | 1. Mouse 3T3 cells. 2. Leukocytes. 3. U2OS cell expressing apelin receptor. 4. Cortical neuronal cultures | (16, 329, 335, 376) |
| XIP | RRLLFYKYVYKRYRAGKQRG | NCX1; reduced activity | Heart cells | (373) |
| ALX40-4C | Ac-rrrrrrrrr-NH_2_ | 1. CXCR4; reduced HIV cell infection; 2. Apelin receptor; block ligand-induced apelin receptor internalisation | 1. T-cells; 2. 293 expressing apelin receptor | (334, 367) |
| CARPs 2 to 6 mers | RR-NH_2_, RW-NH_2_, RRR-NH_2_, RRRRRR-NH_2_, RRRCRW-NH_2_, RCRCWW-NH_2_, RYYRRW-NH_2_ | NMDAR (NR1-NR2a) and/or AMPAR (GluR1); reduced receptor-evoked ionic currents | *Xenopus* oocytes expressing NMDA or AMPA receptors | (1) |
| CARP 6-mers | RRRRWW-NH_2_, RRWWIR-NH_2_, RYYRRW-NH_2_ | VR-1 (TRPV1); reduced activity | *Xenopus* oocytes the expressing VR-1 receptor and DRG neuronal cultures | (2, 209) |
| Dynorphin A 1-17,  Dynorphin A 2-13,  Dynorphin A 1-10 | YGGFLRRIRPKLKWDNQ-NH_2_, GGFLRRIRPKLK-NH_2_,  YGGFLRRIRP-NH_2_ | VR-1 (TRPV1); reduced activity | *Xenopus* oocytes the expressing VR-1 receptor | (2) |
| dRK6 | rrkrrr | VEGFR; reduced VEGF-induced production of TNF-α and IL-6 | Mononuclear cells | (321) |
| TAT,  Penetratin,  R9 | YGRKKRRQRRRG, RQIKIWFQNRRMKWKK, RRRRRRRRR | TNFR, EGFR; reduced cell surface levels | HeLa cell cultures | (229) |
| TAT-JNKI-1D | tdqsrpvqpflnlttprkprpp-rrrqrrkkrG-NH_2_ | Amyloid precursor protein; reduced cell surface levels | Cortical neuronal cultures | (418) |
| PACAP38,  PACAP6-38 | HSDGIFTDSYSRYRKQMAVKKYLAAVLGKRYKQRVKNK-NH_2_,  FTDSYSRYRKQMAVKKYLAAVLGKRYKQRVKNK-NH_2_ | TRPV1; blocked capsaicin-evoked and electrically evoked neuropeptide release | Sensory nerve terminal and cytotrophoblast cells | (183) |
| TAT-NR2B9c | TAT- KLSSIESDV | NMDAR; reduced NR1 subunit cell surface levels | Striatal neuronal cultures | (384) |
| TAT-H11 | TAT-TDRLFMNSIWPG | SR-A; inhibited binding and uptake of acetylated low-density lipoprotein and lipid accumulation | THP-1 monocytic leukemia cell macrophages | (372) |
| TAT-STEP_46_ | TAT-GLQERRGSNVSLTLDM | NMDAR: reduced NR2B and NR1 subunit cell surface levels | Cortical slices, cortical neuronal cultures | (147) |
| Tat-D2_LIL3-29-2_ | TAT-MKSNGSFPVNRRRMD | D1 and D2 receptors; blocked activation induced calcium release | HEK cells expressing D1 and D2 receptors | (112) |
| Tat-Src (40-58) | TAT-PASADGHRGPSAAFVPPAA | NMDAR; reduced NR2B subunit cell surface levels | Amygdala neuronal cultures | (423) |
| TAT-NR2Bct | TAT-NRRRNSKLQHKKY | NMDAR; reduced NMDA calcium influx | Cortical neuronal cultures | (111) |
| TAT-21-40 | TAT-RIPLSKREGIKWQRPRFTRQ | NMDAR; reduced NR1 subunit cell surface levels and NMDA receptor currents | Hippocampal neuronal cultures | (156) |
| TAT-CBD3 | TAT-ARSRLAELRGVPRGL | 1. NMDAR; reduced neuronal cell surface levels, calcium influx and eEPSCs. 2. CaV2.2; reduced cell surface levels, eEPSCs and calcium currents 3. NCX3; reduced cell surface levels | 1. Cortical neuronal cultures. 2. CAD neuronal cell line, mouse spinal cord slices, DRG neurons. 3. Hippocampal neuronal cultures | (100, 101, 433) |
| TAT-L1,  TAT-ct-dis | TAT-YLEWIFKAEEVMLAE,  TAT-NSSFPSIHCSSSWSEE | CaV2.2; reduced potassium induced intracellular calcium influx | DRG neuronal cultures | (381) |
| Myr-tat-CBD3 | TAT-ARSRLAELRGVPRGL | CaV2.2; reduced cell surface levels and calcium influx and currents | DRG neuronal cultures | (406) |
| TAT-3.2-III-IV | TAT-EARRREEKRLRRLERRRRKAQ | CaV3.2; reduced calcium influx and protein levels | DRG neuronal cultures and tissue | (144) |
| TAT-T406 | TAT-IAYSSSETPNRHDML | TRPV1; reduced cell surface levels and function | DRG neuronal cultures and HEK cells expressing TRPV1 | (155) |
| R9-CBD3 | R9-ARSRLAELRGVPRGL | NMDAR; reduced glutamate and NMDA calcium influx | Cortical neuronal cultures | (105) |
| TAT-D1Rc | TAT-LVYLIPHAVGSSEDLKREEAG  GIPKPLEKL | NMDAR; reduced NR1 subunit membrane levels | Membrane fraction from striatal tissue | (374) |
| R9D, R15,  R18,  TAT,  TAT-NR2B9c,  TAT-JNKI-1, PYC36-TAT, BEN1079, R12W8a | rrrrrrrrr-NH2, RRRRRRRRRRRRRRR, RRRRRRRRRRRRRRRRRR,  Ac-GRKKRRQRRRG-NH_2_,  TAT-KLSSIESDV,  TAT-PPRPKRPTTLNLFPQVPRSQDT, TAT-GGLQGRRRQGYQSIKP-NH_2_, RCGRASRCRVRWMRRRRI, WWRRRRWWRRRRWWRRRRWW | Glutamate receptors; reduced glutamic acid induced calcium influx | Cortical neuronal cultures | (15, 16) |
| TAT-4BB | TAT-LHYRDFIPGVAIAA | TLR; reduced TLR induced calcium influx and excitability | DRG neuronal cultures | (396) |
| R9-CBD3-A6K | R9-ARSRLKELRGVPRGL | CaV2.2; reduced cell surface levels and calcium influx and currents | DRG neuronal cultures | (443) |
| RRNY | RRNYRRNY | Cx43 hemichannels; inhibited plasma membrane Cx43 currents and mitochondria Cx43 calcium influx | HeLa cell cultures, cardiac mitochondria | (439) |
| R12,  TAT-NR2B9c | RRRRRRRRRRRR,  TAT-KLSSIESDV | NMDAR; reduce NR2B subunit cell surface levels | Cortical neuronal cultures | (228) |
| t-CNRP1 | TAT-HVTEGSGRYIPRKPF | CaV2.2; reduced cell surface levels and calcium influx | DRG neuronal cultures | (417) |
| TAT-C1aB | TAT-HLSPNKWKW | KV2.1; suppressed potassium currents | CHO cells | (157) |
| t-CSM | TAT-GKMDENQ | NaV1.7; reduced sodium currents and sodium influx | CAD cells, DRG neuronal cultures | (429) |
| TAT-GAP19 | TAT-KQIEIKKFK | Cx43 hemichannels; reduced Cx43 hemichannel opening | Astrocytes in brain slices | (378) |
| TDP-r8 | YrFG-rrrrrrrr-G | NMDAR; binding of peptide to NMDA receptor | Simulated molecular docking studies | (161) |
| W2R4, R6, R8, R16 | WWRRRR, RRRRRR, RRRRRRRR,  RRRRRRRRRRRRRRRR | nAChR; inhibited muscle-type nACh receptor currents | *Xenopus* oocytes and Neuro2a cells expressing different nAChRs | (388) |

At the N-terminus, Ac indicates acetyl and at the C-terminus NH_2_ indicates amide. Lower case single letter code indicates D-isoform of the amino acid. R9 = RRRRRRRRR. TAT = YGRKKRRQRRR or RKKRRQRRR. AMPAR - α-amino-3-hydroxy-5-methyl-4-isoxazolepropionic acid receptor; CAD - Catecholamine A differentiated; CaV2.2 - N-type voltage-gated calcium channel; CaV3.2 - T-type voltage-gated calcium channel; CX43 - Connexin 43; CXCR-4 - C-X-C chemokine receptor type 4; D1 and D2 - Dopamine receptors; DRG - rat dorsal root ganglion; EGFR - Epidermal growth factor receptor; eEPSCs - Evoked excitatory postsynaptic currents; GluR1 - α-amino-3-hydroxy-5-methyl-4-isoxazolepropionic acid receptor; HIV - human immunodeficiency virus; IL-6 - Interleukin-6; KV2.1 - Voltage-gated potassium channel; NaV1.7; Voltage-gated sodium channel; Myr - N-myristoyl; NCX - sodium-calcium exchanger; nAChR -nicotinic acetylcholine receptor; NMDAR - N-methyl-D-aspartate receptor; PDGF - Platelet-derived growth factor; SR-A - Class A scavenger receptor; TRVP1 - transient receptor potential cation channel subfamily V member 1; TNF-α - Tumor necrosis factor-α; TNFR - Tumor necrosis factor-α receptor; TLR - Toll-like receptors; VEGFR - Vascular endothelial growth factor receptor; VR-1 - vanilloid receptor 1.

**Supplementary table 3** Guanidine moiety containing agents with neuroprotective or neuroactive properties.

| **Agent** | **Physiological charge** | **Neuronal injury model** | **References** |
| --- | --- | --- | --- |
| Substituted guanidine derivatives | +1 | Excitotoxicity, stroke | (232, 233, 410) |
| Metformin | +2 | Excitotoxicity, AD, PD, GCI, stroke, SCI, HIE, TBI, ICH, pain | (370, 388, 390, 391, 395, 397, 441, 442, 432) |
| Phenformin | +2 | Excitotoxicity | (408) |
| Aminoguanidine | +1 | Stroke, AD, PD HIE, pain | (399, 412, 414, 428, 431) |
| Agmatine | +2 | Excitotoxicity, stroke, GCI, SCI, TBI, HIE, pain | (383, 394, 398, 404, 407) |
| Creatine | 0 | Excitotoxicity, stroke, GCI, AD, HD, PD, ALS, SCI, TBI, HIE | (380, 387, 392, 400, 403, 405, 420, 430, 436-438) |
| 2-iminobiotin (cyclic guanidine) | 0 | HIE | (382, 419, 440) |
| Amiloride | 0 | Stroke, PD, MS, SCI, pain | (371, 393, 411, 426, 427) |
| L-NAME | +2 | Excitotoxicity, stroke, GCI, HIE, SCI, TBI | (375, 386, 409, 413, 416, 422, 434) |
| L-NNA | 0 | Excitotoxicity, HIE, stroke | (361, 401, 424) |
| L-arginine | +1 | Stroke, SCI | (379, 385, 402, 425) |
| Tetrodotoxin | +1 | GCI, OGD | (377, 435) |

L-NAME - Nω-Nitro-L-arginine methyl ester hydrochloride or N(G)-Nitro-L-arginine methyl ester; L-NNA - Nω-Nitro-L-arginine or N(G)-Nitro-L-arginine; AD - Alzheimer's disease; ALS - amyotrophic lateral sclerosis; EAE - Experimental autoimmune encephalomyelitis; GCI - global cerebral ischaemia; HIE - hypoxia-ischaemia encephalopathy; HD – Huntington’s disease; ICH - intracerebral haemorrhage; MS – multiple sclerosis; PD - Parkinson’s disease; SCI - spinal cord injury; stroke - ischaemic stroke; TBI - traumatic brain injury. Physiological charge - as reported by Drugbank (<https://www.drugbank.ca/>).

**Supplementary References**

1. Ferrer-Montiel AV, Merino JM, Blondelle SE, Perez-Payà E, Houghten RA, Montal M. Selected peptides targeted to the NMDA receptor channel protect neurons from excitotoxic death. Nat Biotechnol. (1998) 16:286–91. doi: 10.1038/nbt0398-286

2. Planells-Cases R, Aracil A, Merino JM, Gallar J, Pérez-Payá E, Belmonte C, et al. Arginine-rich peptides are blockers of VR-1 channels with analgesic activity. FEBS Lett. (2000) 481:131–36. doi: 10.1016/S0014-5793(00)01982-7

15. Meloni BP, Brookes LM, Clark VW, Cross JL, Edwards AB, Anderton RS, et al. Poly-arginine and arginine-rich peptides are neuroprotective in stroke models. J Cereb Blood Flow Metab. (2015) 35:993–1004. doi: 10.1038/jcbfm.2015.11

16. Meloni BP, Milani D, Cross JL, Clark VW, Edwards AB, Anderton RS, et al. Assessment of the neuroprotective effects of arginine-rich protamine peptides poly-arginine peptides (R12-cylic R22) and arginine-tryptophan containing peptides following in vitro excitotoxicity and/or permanent middle cerebral artery occlusion in rats. Neuromol Med. (2017) 19:271–85. doi: 10.1007/s12017-017-8441-2

100. Liu XJ, Gingrich JR, Vargas-Caballero M, Dong YN, Sengar A, Beggs S, et al. Treatment of inflammatory and neuropathic pain by uncoupling Src from the NMDA receptor complex. Nat Med. (2008) 14:1325–32. doi: 10.1038/nm.1883

101. Chambers JW, Howard S, LoGrasso PV. Blocking c-Jun N-terminal kinase (JNK) translocation to the mitochondria prevents 6-hydroxydopamine induced toxicity in vitro and in vivo. J Biol Chem. (2013) 288:1079–87. doi: 10.1074/jbc.M112.421354

105. Piekarz AD, Due MR, Khanna M, Wang B, Ripsch MS, Wang R, et al. CRMP-2 peptide mediated decrease of high and low voltage-activated calcium channels attenuation of nociceptor excitability and anti-nociception in a model of AIDS therapy-induced painful peripheral neuropathy. Mol Pain. (2012) 8:2–19. doi: 10.1186/1744-8069-8-54

112. McQueen J, Ryan TJ, McKay S, Marwick K, Baxter P, Carpanini SM, et al. Pro-deathNMDA receptor signaling is promoted by the GluN2B C-terminus independently of Dapk1. Elife. (2017) 6:e17161. doi: 10.7554/eLife.17161.024

113. Tu W, Xu X, Peng L, Zhong X, Zhang W, Soundarapandian MM, et al. DAPK1 interaction with NMDA receptor NR2B subunits mediates brain damage in stroke. Cell. (2010) 140:222–34. doi: 10.1016/j.cell.2009.12.055

144. García-Caballero A, Gadotti VM, Stemkowski P, Weiss N, Souza IA, Hodgkinson V, et al. The deubiquitinating enzyme USP5 modulates neuropathic and inflammatory pain by enhancing Cav32 channel activity. Neuron. (2014) 83:1144–58. doi: 10.1016/j.neuron.2014.07.036

147. Xu J, Kurup P, Zhang Y, Goebel-Goody SM, Wu PH, Hawasli AH, et al. Extrasynaptic NMDA receptors couple preferentially to excitotoxicity via calpain-mediated cleavage of STEP. J Neurosci. (2009) 29:9330–43. doi: 10.1523/JNEUROSCI.2212-09.2009

155. Liu J, Du J, Yang Y, Wang Y. Phosphorylation of TRPV1 by cyclin-dependent kinase 5 promotes TRPV1 surface localization leading to inflammatory thermal hyperalgesia. Exp Neurol. (2015) 273:253–62. doi: 10.1016/j.expneurol.2015.09.005

156. Zhang Y, Su P, Liang P, Liu T, Liu X, Liu XY, et al. The DREAM protein negatively regulates the NMDA receptor through interaction with the NR1 subunit. J Neurosci. (2010) 30:7575–86. doi: 10.1523/JNEUROSCI.1312-10.2010

157. Yeh CY, Bulas AM, Moutal A, Saloman JL, Hartnett KA, Anderson CT, et al. Targeting a potassium channel/syntaxin interaction ameliorates cell death in ischemic stroke. J Neurosci. (2017) 37:5648–58. doi: 10.1523/JNEUROSCI.3811-16.2017

161. Kashkin VA, Shekunova EV, Titov MI, Eliseev II, Gureev MA, Porozov YB, et al. A new tridecapeptide with an octaarginine vector has analgesic therapeutic potential and prevents morphine-induced tolerance. Peptides. (2018) 99:61–9. doi: 10.1016/j.peptides.2017.11.011

183. Reglodi D, Borzsei R, Bagoly T, Boronkai A, Racz B, Tamas A, et al. Agonistic behavior of PACAP6–38 on sensory nerve terminals and cytotrophoblast cells. J Mol Neurosci. (2008) 36:270–8. doi: 10.1007/s12031-008-9089-z

209. Himmel HM, Kiss T, Borvendeg SJ, Gillen C, Illes P. The arginine-rich hexapeptide R4W2 is a stereoselective antagonist at the vanilloid receptor 1: a Ca2+ imaging study in adult rat dorsal root ganglion neurons. J Pharmacol Exp Ther. (2002) 301:981–6. doi: 10.1124/jpet.301.3.981

228. MacDougall G, Anderton RS, Edwards AB, Knuckey NW, Meloni BP. The Neuroprotective peptide poly-arginine-12 (R12) reduces cell surface levels of NMDA NR2B receptor subunit in cortical neurons; investigation into the involvement of endocytic mechanisms. J Mol Neurosci. (2017) 61:235–46. doi: 10.1007/s12031-016-0861-1

229. Fotin-Mleczek M, Welte S, Mader O, Duchardt F, Fischer R, Hufnagel H, et al. Cationic cell-penetrating peptides interfere with TNF signalling by induction of TNF receptor internalization. J Cell Sci. (2005) 118:3339–51. doi: 10.1242/jcs.02460

232. Keana JF, McBurney RN, Scherz MW, Fischer JB, Hamilton PN, Smith SM, et al. Synthesis and characterization of a series of diarylguanidines that are noncompetitive N-methyl-D-aspartate receptor antagonists with neuroprotective properties. Proc Natl Acad Sci USA. (1989) 86:5631–35. doi: 10.1073/pnas.86.14.5631

233. Goldin SM, Subbarao K, Sharma R, Knapp AG, Fischer JB, Daly D, et al. Neuroprotective use-dependent blockers of Na+ and Ca2+ channels controlling presynaptic release of glutamate. Ann N Y Acad Sci. (1995) 765:210–29. doi: 10.1111/j.1749-6632.1995.tb16578.x

321. Yoo SA, Bae DG, Ryoo JW, Kim HR, Park GS, Cho CS, et al. Arginine-rich anti-vascular endothelial growth factor (anti-VEGF) hexapeptide inhibits collagen-induced arthritis and VEGF-stimulated productions of TNF-alpha and IL-6 by human monocytes. J Immunol. (2005) 174:5846–55. doi: 10.4049/jimmunol.174.9.5846

329. Zhang T, Garstka MA, Li K. The controversial C5a receptor C5aR2: its role in health and disease. J Immunol Res. (2017) 2017:8193932. doi: 10.1155/2017/8193932

334. Zhou N, Zhang X, Fan X, Argyris E, Fang J, Acheampong E, et al. The N-terminal domain of APJ a CNS-based coreceptor for HIV-1 is essential for its receptor function and coreceptor activity. Virology. (2003) 317:84–94. doi: 10.1016/S0042-6822(03)00638-X

335. Le Gonidec S, Chaves-Almagro C, Bai Y, Kang HJ, Smith A, Wanecq E, et al. Protamine is an antagonist of apelin receptor and its activity is reversed by heparin. FASEB J. (2017) 31:2507–19. doi: 10.1096/fj.201601074R

367. Doranz BJ, Grovit-Ferbas K, Sharron MP, Mao SH, Goetz MB, Daar ES, et al. Safe use of the CXCR4 inhibitor ALX40–4C in humans. AIDS Res Hum Retroviruses. (2001) 17:475–86. doi: 10.1089/08892220151126508

361. Dawson VL, Dawson TM, London ED, Bredt DS, Snyder SH. Nitric oxide mediates glutamate neurotoxicity in primary cortical cultures. Proc Natl Acad Sci USA. (1991) 88:6368–71. doi: 10.1073/pnas.88.14.6368

370. Ashabi G, Khodagholi F, Khalaj L, Goudarzvand M, Nasiri M. Activation of AMP-activated protein kinase by metformin protects against global cerebral ischemia in male rats: interference of AMPK/PGC-1a pathway. Metab Brain Dis. (2014) 29:47–58. doi: 10.1007/s11011-013-9475-2

371. Sepehrdad R, Chander PN, Oruene A, Rosenfeld L, Levine S, Stier CT Jr. Amiloride reduces stroke and renal injury in stroke-prone hypertensive rats. Am J Hypertens. (2003) 16:312–8. doi: 10.1016/S0895-7061(03)00006-2

372. Wang X, Zheng Y, Xu Y, Ben J, Gao S, Zhu X, et al. A novel peptide binding to the cytoplasmic domain of class A scavenger receptor reduces lipid uptake in THP-1 macrophages. Biochim Biophys Acta. (2009) 1791:76–83. doi: 10.1016/j.bbalip.2008.10.011

373. Chin TK, Spitzer KW, Philipson KD, Bridge JH. The effect of exchanger inhibitory peptide (XIP) on sodium-calcium exchange current in guinea pig ventricular cells. Circ Res. (1993) 72:497–503. doi: 10.1161/01.RES.72.3.497

374. Song L, Zhang Z, Hu R, Cheng J, Li L, Fan Q, et al. Targeting the D1-Nmethyl- D-aspartate receptor complex reduces L-dopa-induced dyskinesia in 6-hydroxydopamine-lesioned Parkinson’s rats. Drug Des Devel Ther. (2016) 10:547–55. doi: 10.2147/DDDT.S93487

375. Abd El-Aal SA, El-Sawalhi MM, Seif-El-Nasr M, Kenawy SA. Effect of celecoxib and L-NAME on global ischemia-reperfusion injury in the rat hippocampus. Drug Chem Toxicol. (2013) 36:385–95. doi: 10.3109/01480545.2012.749270

376. Huang JS, Nishimura J, Huang SS, Deuel TF. Protamine inhibits platelet derived growth factor receptor activity but not epidermal growth factor activity. J Cell Biochem. (1984) 26:205–20. doi: 10.1002/jcb.240260402

377. Lysko PG, Webb CL, Yue TL, Gu JL, Feuerstein G. Neuroprotective effects of tetrodotoxin as a Na+ channel modulator and glutamate release inhibitor in cultured rat cerebellar neurons and in gerbil global brain ischemia. Stroke. (1994) 25:2476–82. doi: 10.1161/01.STR.25.12.2476

378. Walrave L, Pierre A, Albertini G, Aourz N, De Bundel D, Van Eeckhaut A, et al. Inhibition of astroglial connexin43 hemichannels with TAT-Gap19 exerts anticonvulsant effects in rodents. Glia. (2018) 66:1788–804. doi: 10.1002/glia.23341

379. Koga Y, Ishibashi M, Ueki I, Yatsuga S, Fukiyama R, Akita Y, et al. Effects of L-arginine on the acute phase of strokes in three patients with MELAS. Neurology. (2002) 58:827–8. doi: 10.1212/WNL.58.5.827

380. Lensman M, Korzhevskii DE, Mourovets VO, Kostkin VB, Izvarina N, Perasso L, et al. Intracerebroventricular administration of creatine protects against damage by global cerebral ischemia in rat. Brain Res. (2006) 1114:187–94. doi: 10.1016/j.brainres.2006.06.103

381. Wilson SM, Schmutzler BS, Brittain JM, Dustrude ET, Ripsch MS, Pellman JJ, et al. Inhibition of transmitter release and attenuation of anti-retroviral associated and tibial nerve injury-related painful peripheral neuropathy by novel synthetic Ca2+ channel peptides. J Biol Chem. (2012) 287:35065–77. doi: 10.1074/jbc.M112.378695

382. Peeters-Scholte C, Koster J, Veldhuis W, van den Tweel E, Zhu C, Kops N, et al. Neuroprotection by selective nitric oxide synthase inhibition at 24 hours after perinatal hypoxia-ischemia. Stroke. (2002) 33:2304–10. doi: 10.1161/01.STR.0000028343.25901.09

383. Gilad GM, Salame K, Rabey JM, Gilad VH. Agmatine treatment is neuroprotective in rodent brain injury models. Life Sci. (1996) 58:41–6. doi: 10.1016/0024-3205(95)02274-0

384. Fan J, Cowan CM, Zhang LY, Hayden MR, Raymond LA. Interaction of postsynaptic density protein-95 with NMDA receptors influences excitotoxicity in the yeast artificial chromosome mouse model of Huntington’s disease. J Neurosci. (2009) 29:10928–38. doi: 10.1523/JNEUROSCI.2491-09.2009

385. Yüceer N, Attar A, Sargon MF, Egemen N, Türker RK, Demirel E. The early protective effects of L-arginine and Ng-nitro-L-arginine methyl ester after experimental acute spinal cord injury A light and electron microscopic study. J Clin Neurosci. (2000) 7:238–43. doi: 10.1054/jocn.1999.0210

386. Mésenge C, Verrecchia C, Allix M, Boulu RR, Plotkine M. Reduction of the neurological deficit in mice with traumatic brain injury by nitric oxide synthase inhibitors. J Neurotrauma. (1996) 13:11–16. doi: 10.1089/neu.1996.13.209

387. Adcock KH, Nedelcu J, Loenneker T, Martin E, Wallimann T, Wagner BP. Neuroprotection of creatine supplementation in neonatal rats with transient cerebral hypoxia-ischemia. Dev Neurosci. (2002) 24:382–8. doi: 10.1159/000069043

388. Wang C, Liu C, Gao K, Zhao H, Zhou Z, Shen Z, et al. Metformin preconditioning provide neuroprotection through enhancement of autophagy and suppression of inflammation and apoptosis after spinal cord injury. Biochem Biophys Res Commun. (2016) 477:534–40. doi: 10.1016/j.bbrc.2016.05.148

389. Lebedev DS, Kryukova EV, Ivanov IA, Egorova NS, Timofeev ND, Spirova EN, et al. Oligoarginine peptides, a new family of nicotinic acetylcholine receptor inhibitors. Mol Pharmacol. (2019) 96:664–73. doi: 10.1124/mol.119.117713

390. Qi B, Hu L, Zhu L, Shang L, Wang X, Liu N, et al. Metformin attenuates neurological deficit after intracerebral hemorrhage by inhibiting apoptosis oxidative stress and neuroinflammation in rats. Neurochem Res. (2017) 42:2912–20. doi: 10.1007/s11064-017-2322-9

391. Zhou C, Sun R, Zhuang S, Sun C, Jiang Y, Cui Y, et al. Metformin prevents cerebellar granule neurons against glutamate-induced neurotoxicity. Brain Res Bull. (2016) 121:241–45. doi: 10.1016/j.brainresbull.2016.02.009

392. Dupuis L, Oudart H, René F, Gonzalez de Aguilar JL, Loeffler JP. Evidence for defective energy homeostasis in amyotrophic lateral sclerosis: benefit of a high-energy diet in a transgenic mouse model. Proc Natl Acad Sci USA. (2004) 101:11159–64. doi: 10.1073/pnas.0402026101

393. Rocha-González HI, Herrejon-Abreu EB, López-Santillán FJ, García-López BE, Murbartián J, Granados-Soto V. Acid increases inflammatory pain in rats: effect of local peripheral ASICs inhibitors. Eur J Pharmacol. (2009) 603:56–61. doi: 10.1016/j.ejphar.2008.12.017

394. Feng Y, Piletz JE, Leblanc MH. Agmatine suppresses nitric oxide production and attenuates hypoxic-ischemic brain injury in neonatal rats. Pediatr Res. (2002) 52:606–11. doi: 10.1203/00006450-200210000-00023

395. Hill JL, Kobori N, Zhao J, Rozas NS, Hylin MJ, Moore AN, et al. Traumatic brain injury decreases AMP-activated protein kinase activity and pharmacological enhancement of its activity improves cognitive outcome. J Neurochem. (2016) 139:106–19. doi: 10.1111/jnc.13726

396. Allette YM, Kim Y, Randolph AL, Smith JA, Ripsch MS, White FA. Decoy peptide targeted to Toll-IL-1R domain inhibits LPS and TLR4-active metabolite morphine-3 glucuronide sensitization of sensory neurons. Sci Rep. (2017) 7:3741. doi: 10.1038/s41598-017-03447-9

397. Katila N, Bhurtel S, Shadfar S, Srivastav S, Neupane S, Ojha U, et al. Metformin lowers a-synuclein phosphorylation and upregulates neurotrophic factor in the MPTP mouse model of Parkinson’s disease. Neuropharmacology. (2017) 125:396–407. doi: 10.1016/j.neuropharm.2017.08.015

398. Horváth G, Kékesi G, Dobos I, Szikszay M, Klimscha W, Benedek G. Effect of intrathecal agmatine on inflammation-induced thermal hyperalgesia in rats. Eur J Pharmacol. (1999) 368:197–204. doi: 10.1016/S0014-2999(99)00060-6

399. Lu G, Su RB, Li J, Qin BY. Modulation by alpha-difluoromethyl-ornithine and aminoguanidine of pain threshold morphine analgesia and tolerance. Eur J Pharmacol. (2003) 478:139–44. doi: 10.1016/j.ejphar.2003.08.048

400. Andres RH, Huber AW, Schlattner U, Pérez-Bouza A, Krebs SH, Seiler RW, et al. Effects of creatine treatment on the survival of dopaminergic neurons in cultured fetal ventral mesencephalic tissue. Neuroscience. (2005) 133:701–13. doi: 10.1016/j.neuroscience.2005.03.004

401. Carreau A, Duval D, Poignet H, Scatton B, Vigé X, Nowicki JP. Neuroprotective efficacy of N omega-nitro-L-arginine after focal cerebral ischemia in the mouse and inhibition of cortical nitric oxide synthase. Eur J Pharmacol. (1994) 256:241–9. doi: 10.1016/0014-2999(94)90549-5

402. Harston GW, Sutherland BA, Kennedy J, Buchan AM. The contribution of L-arginine to the neurotoxicity of recombinant tissue plasminogen activator following cerebral ischemia: a review of rtPA neurotoxicity. J Cereb Blood Flow Metab. (2010) 30:1804–16. doi: 10.1038/jcbfm.2010.149

403. Sullivan PG, Geiger JD, Mattson MP, Scheff SW. Dietary supplement creatine protects against traumatic brain injury. Ann Neurol. (2000) 48:723–9. doi:10.1002/1531-8249(200011)48:5<723::AID-ANA5>3.0.CO;2-W

404. Kim JY, Lee YW, Kim JH, Lee WT, Park KA, Lee JE. Agmatine attenuates brain edema and apoptotic cell death after traumatic brain injury. J Korean Med Sci. (2015) 30:943–52. doi: 10.3346/jkms.2015.30.7.943

405. Zhu S, Li M, Figueroa BE, Liu A, Stavrovskaya IG, Pasinelli P, et al. Prophylactic creatine administration mediates neuroprotection in cerebral ischemia in mice. J Neurosci. (2004) 24:5909–12. doi: 10.1523/JNEUROSCI.1278-04.2004

406. François-Moutal L, Wang Y, Moutal A, Cottier KE, Melemedjian OK, Yang X, et al. A membrane-delimited N-myristoylated CRMP2 peptide aptamer inhibits CaV2.2 trafficking and reverses inflammatory and postoperative pain behaviors. Pain. (2015) 156:1247–64. doi: 10.1097/j.pain.0000000000000147

407. Yu CG, Marcillo AE, Fairbanks CA, Wilcox GL, Yezierski RP. Agmatine improves locomotor function and reduces tissue damage following spinal cord injury. Neuroreport. (2000) 11:3203–07. doi: 10.1097/00001756-200009280-00031

408. Lee J, Chan SL, Lu C, Lane MA, Mattson MP. Phenformin suppresses calcium responses to glutamate and protects hippocampal neurons against excitotoxicity. Exp Neurol. (2002) 175:161–7. doi: 10.1006/exnr.2002.7864

409. Wainwright MS, Grundhoefer D, Sharma S, Black SM. A nitric oxide donor reduces brain injury and enhances recovery of cerebral blood flow after hypoxia-ischemia in the newborn rat. Neurosci Lett. (2007) 415:124–9. doi: 10.1016/j.neulet.2007.01.019

410. Kirk CJ, Reddy NL, Fischer JB, Wolcott TC, Knapp AG, McBurney RN. In vitro neuroprotection by substituted guanidines with varying affinities for the N-methyl-D-aspartate receptor ionophore and for sigma sites. J Pharmacol Exp Ther. (1994) 271:1080–5.

411. Arias RL, Sung ML, Vasylyev D, Zhang MY, Albinson K, Kubek K, et al. Amiloride is neuroprotective in an MPTP model of Parkinson’s disease. Neurobiol Dis. (2008) 31:334–41. doi: 10.1016/j.nbd.2008.05.008

412. Díaz A, Rojas K, Espinosa B, Chávez R, Zenteno E, Limón D, et al. Aminoguanidine treatment ameliorates inflammatory responses and memory impairment induced by amyloid-beta 25–35 injection in rats. Neuropeptides. (2014) 48:153–9. doi: 10.1016/j.npep.2014.03.002

413. Fujisawa H, Dawson D, Browne SE, MacKay KB, Bullock R, McCulloch J. Pharmacological modification of glutamate neurotoxicity in vivo. Brain Res. (1993) 629:73–8. doi: 10.1016/0006-8993(93)90483-4

414. Iadecola C, Zhang F, Xu X. Inhibition of inducible nitric oxide synthase ameliorates cerebral ischemic damage. Am J Physiol. (1995) 268:286–92. doi: 10.1152/ajpregu.1995.268.1.R286

415. Jiang T, Yu JT, Zhu XC, Wang HF, Tan MS, Cao L, et al. Acute metformin preconditioning confers neuroprotection against focal cerebral ischaemia by pre-activation of AMPK-dependent autophagy. Br J Pharmacol. (2014) 171:3146–57. doi: 10.1111/bph.12655

416. Pramila B, Kalaivani P, Anita A, Saravana Babu C. L-NAME combats excitotoxicity and recuperates neurological deficits in MCAO/R rats. Pharmacol Biochem Behav. (2015) 135:246–53. doi: 10.1016/j.pbb.2015.06.006

417. Moutal A, Wang Y, Yang X, Ji Y, Luo S, Dorame A, et al. Dissecting the role of the CRMP2-neurofibromin complex on pain behaviors. Pain. (2017) 158:2203–21. doi: 10.1097/j.pain.0000000000001026

418. Colombo A, Repici M, Pesaresi M, Santambrogio S, Forloni G, Borsello T. The TAT-JNK inhibitor peptide interferes with beta amyloid protein stability. Cell Death Differ. (2007) 14:1845–48. doi: 10.1038/sj.cdd.4402202

419. Nijboer CH, Groenendaal F, Kavelaars A, Hagberg HH, van Bel F, Heijnen CJ. Gender-specific neuroprotection by 2-iminobiotin after hypoxia-ischemia in the neonatal rat via a nitric oxide independent pathway. J Cereb Blood Flow Metab. (2007) 27:282–92. doi: 10.1038/sj.jcbfm.9600342

420. Malcon C, Kaddurah-Daouk R, Beal MF. Neuroprotective effects of creatine administration against NMDA and malonate toxicity. Brain Res. (2000) 860:195–8. doi: 10.1016/S0006-8993(00)02038-2

421. Doranz BJ, Grovit-Ferbas K, Sharron MP, Mao SH, Goetz MB, Daar ES, et al. A small-molecule inhibitor directed against the chemokine receptor CXCR4 prevents its use as anHIV-1 coreceptor. J Exp Med. (1997) 186:1395–400. doi: 10.1084/jem.186.8.1395

422. Iadecola C, Pelligrino DA, Moskowitz MA, Lassen NA. Nitric oxide synthase inhibition and cerebrovascular regulation. J Cereb Blood Flow Metab. (1994) 114:175–92. doi: 10.1038/jcbfm.1994.25

423. Sinai L, Duffy S, Roder JC. Src inhibition reduces NR2B surface expression and synaptic plasticity in the amygdala. Learn Mem. (2010) 17:364–71. doi: 10.1101/lm.1765710

424. Trifiletti RR. Neuroprotective effects of NG-nitro-L-arginine in focal stroke in the 7-day old rat. Eur J Pharmacol. (1992) 218:197–8. doi: 10.1016/0014-2999(92)90168-4

425. Morikawa E, Huang Z, Moskowitz MA. L-arginine decreases infarct size caused by middle cerebral arterial occlusion in SHR. Am J Physiol. (1992) 263:1632–5 doi: 10.1152/ajpheart.1992.263.5.H1632

426. Arun T, Tomassini V, Sbardella E, de Ruiter MB, Matthews L, Leite MI, et al. Targeting ASIC1 in primary progressive multiple sclerosis: evidence of neuroprotection with amiloride. Brain. (2013) 136:106–15. doi: 10.1093/brain/aws325

427. Fehlings MG, Agrawal S. Role of sodium in the pathophysiology of secondary spinal cord injury. Spine (Phila Pa 1976). (1995) 20:2187–91. doi: 10.1097/00007632-199510001-00002

428. Hsu YC, Chang YC, Lin YC, Sze CI, Huang CC, Ho CJ. Cerebral microvascular damage occurs early after hypoxia-ischemia via nNOS activation in the neonatal brain. J Cereb Blood Flow Metab. (2014) 34:668–76. doi: 10.1038/jcbfm.2013.244

429. François-Moutal L, Dustrude ET, Wang Y, Brustovetsky T, Dorame A, Ju W, et al. Inhibition of the Ubc9 E2 SUMO conjugating enzyme-CRMP2 interaction decreases NaV1.7 currents and reverses experimental neuropathic pain. Pain. (2018) 159:2115–27. doi: 10.1097/j.pain.0000000000001294

430. Brewer GJ, Wallimann TW. Protective effect of the energy precursor creatine against toxicity of glutamate and beta-amyloid in rat hippocampal neurons. J Neurochem. (2000) 74:1968–78. doi: 10.1046/j.1471-4159.2000.0741968.x

431. Yadav S, Gupta SP, Srivastava G, Srivastava PK, Singh MP. Role of secondary mediators in caffeine-mediated neuroprotection in maneb- and paraquat-induced Parkinson’s disease phenotype in the mouse. Neurochem Res. (2012) 37:875–84. doi: 10.1007/s11064-011-0682-0

432. Melemedjian OK, Asiedu MN, Tillu DV, Sanoja R, Yan J, Lark A, et al. Targeting adenosine monophosphate-activated protein kinase (AMPK) in preclinical models reveals a potential mechanism for the treatment of neuropathic pain. Mol Pain. (2011) 7:70. doi: 10.1186/1744-8069-7-70

433. Brustovetsky T, Pellman JJ, Yang XF, Khanna R, Brustovetsky N. Collapsin response mediator protein 2 (CRMP2) interacts with N-methyl-D-aspartate (NMDA) receptor and Na+/Ca2+ exchanger and regulates their functional activity. J Biol Chem. (2014) 289:7470–82. doi: 10.1074/jbc.M113.518472

434. Sharma HS, Badgaiyan RD, Alm P, Mohanty S, Wiklund L. Neuroprotective effects of nitric oxide synthase inhibitors in spinal cord injury-induced

pathophysiology and motor functions: an experimental study in the rat. Ann N Y Acad Sci. (2005) 1053:422–34. doi: 10.1196/annals.1344.037

435. Weber ML, Taylor CP. Damage from oxygen and glucose deprivation in hippocampal slices is prevented by tetrodotoxin lidocaine and phenytoin without blockade of action potentials. Brain Res. (1994) 664:167–77. doi: 10.1016/0006-8993(94)91967-4

436. Prass K, Royl G, Lindauer U, Freyer D, Megow D, Dirnagl U, et al. Improved reperfusion and neuroprotection by creatine in a mouse model of stroke. J Cereb Blood Flow Metab. (2007) 27:452–9. doi: 10.1038/sj.jcbfm.9600351

437. Dedeoglu A, Kubilus JK, Yang L, Ferrante KL, Hersch SM, Beal MF, et al. Creatine therapy provides neuroprotection after onset of clinical symptoms in Huntington’s disease transgenic mice. J Neurochem. (2003) 85:1359–67. doi: 10.1046/j.1471-4159.2003.01706.x

438. Hausmann ON, Fouad K, Wallimann T, Schwab ME. Protective effects of oral creatine supplementation on spinal cord injury in rats. Spinal Cord. (2002) 40:449–56. doi: 10.1038/sj.sc.3101330

439. Gadicherla AK, Wang N, Bulic M, Agullo-Pascual E, Lissoni A, De Smet M, et al. Mitochondrial Cx43 hemichannels contribute to mitochondrial calcium entry and cell death in the heart. Basic Res Cardiol. (2017) 112:27. doi: 10.1007/s00395-017-0618-1

440. Bjorkman ST, Ireland Z, Fan X, van der Wal WM, Roes KC, Colditz PB, et al. Short-term dose-response characteristics of 2-iminobiotin immediately post-insult in the neonatal piglet after hypoxia-ischemia. Stroke. (2013) 44:809–11. doi: 10.1161/STROKEAHA.112.677922

441. Ou Z, Kong X, Sun X, He X, Zhang L, Gong Z, et al. Metformin treatment prevents amyloid plaque deposition and memory impairment in APP/PS1 mice. Brain Behav Immun. (2018) 69:351–63. doi: 10.1016/j.bbi.2017.12.009

442. Fang M, Jiang H, Ye L, Cai C, Hu Y, Pan S, et al. Metformin treatment after the hypoxia-ischemia attenuates brain injury in newborn rats. Oncotarget. (2017) 8:75308–25. doi: 10.18632/oncotarget.20779

443. Xie JY, Chew LA, Yang X, Wang Y, Qu C, Wang Y, et al. Sustained relief of ongoing experimental neuropathic pain by a CRMP2 peptide aptamer with low abuse potential. Pain. (2016) 157:2124–40. doi: 10.1097/j.pain.0000000000000628
